# Analysis of the PEBP gene family and identification of a novel *FLOWERING LOCUS T* orthologue in sugarcane

**DOI:** 10.1093/jxb/erab539

**Published:** 2021-12-10

**Authors:** Julien Venail, Paulo Henrique da Silva Santos, Joao Ricardo Manechini, Leonardo Cardosos Alves, Maximiliano Scarpari, Thais Falcão, Elisson Romanel, Michael Brito, Renato Vicentini, Luciana Pinto, Stephen Derek Jackson

**Affiliations:** 1 School of Life Sciences, University of Warwick, Gibbet Hill, Coventry CV4 7AL, UK; 2 Centro de Cana, Instituto Agronômico de Campinas (IAC), Ribeirão Preto, São Paulo, Brazil; 3 Instituto de Biologia, Universidade Estadual de Campinas (UNICAMP), Campinas, São Paulo, Brazil; 4 SUPERBAC Biotechnology Solutions, Londrina, Paraná, Brazil; 5 Departamento de Biotecnologia, Escola de Engenharia de Lorena (EEL), Universidade de São Paulo (USP), São Paulo, Brazil; 6 Instituto de Ciência e Tecnologia, Universidade Federal de São Paulo (UNIFESP), São José dos Campos, São Paulo, Brazil; 7 Indian Institute of Science, India

**Keywords:** Flowering, Flowering locus T, meristem, PEBP, photoperiod, sugarcane

## Abstract

Sugarcane (*Saccharum* spp.) is an important economic crop for both sugar and biomass, the yields of which are negatively affected by flowering. The molecular mechanisms controlling flowering in sugarcane are nevertheless poorly understood. RNA-seq data analysis and database searches have enabled a comprehensive description of the PEBP gene family in sugarcane. It is shown to consist of at least 13 *FLOWERING LOCUS T* (*FT*)-like genes, two *MOTHER OF FT AND TFL* (*MFT*)*-*like genes, and four *TERMINAL FLOWER* (*TFL*)-like genes. As expected, these genes all show very high homology to their corresponding genes in *Sorghum*, and also to *FT*-like, *MFT-*like, and *TFL*-like genes in maize, rice, and Arabidopsis. Functional analysis in Arabidopsis showed that the sugarcane *ScFT3* gene can rescue the late flowering phenotype of the Arabidopsis *ft-10* mutant, whereas *ScFT5* cannot. High expression levels of *ScFT3* in leaves of short day-induced sugarcane plants coincided with initial stages of floral induction in the shoot apical meristem as shown by histological analysis of meristem dissections. This suggests that *ScFT3* is likely to play a role in floral induction in sugarcane; however, other sugarcane *FT*-like genes may also be involved in the flowering process.

## Teaser

We identify a *FLOWERING LOCUS T*(*FT*) gene, out of a family of 13 *FT-*like genes in sugarcane, that is expressed in photoperiodically inductive conditions and functions as a floral inducer.

## Introduction

Sugarcane (*Saccharum* spp.) is a major economic crop grown in 107 different countries, with global production in 2019 being 1.95 billion metric tonnes (https://www.statista.com/statistics/249604/sugar-cane-production-worldwide/). As well as accounting for ~80% of global sugar production, sugarcane is also used in the production of bioethanol and as a biomass crop for biofuels. As sugarcane is one of the most efficient photosynthetic plants, it can provide cost-competitive first- and second-generation bioethanol from the extracted sugar and the lignocellulosic biomass, respectively (Menz *et al.*, 1969; Tew and Cobill, 2008; Dias *et al.*, 2011). The waste from this process (the lignin biomass and cane waste) is either burnt to generate electricity or made into biomass pellets for export to other countries.

Sugarcane is a perennial grass in the same family as maize, wheat, rice, and *Sorghum*. It is highly polyploid (≥8), aneuploid, and heterogeneous, with a large number of chromosomes (2*n*=70–200) thus, whilst sugarcane produces seed, vegetative propagation by stem cuttings (seed cane) is used in order to preserve desired traits (Garsmeur *et al.*, 2018). Conventional breeding in sugarcane is very difficult (taking up to 13 years to produce a new variety) because commercial sugarcane varieties are complex hybrids derived from interspecific hybridizations between *Saccharum officinarum* L. (high sugar and low fibre content) and *Saccharum spontaneum* L. (very high fibre and very low sugar content). The initial *S. officinarum×S. spontaneum* hybrids were backcrossed several times to *S. officinarum* to recover the high sugar content. As a result, commercial sugarcane varieties have ~80% of the *S. officinarum* chromosomes, 10% from *S. spontaneum*, and 10% recombinant chromosomes from both species (Moore and Botha, 2013). Recently, to achieve high-yielding biomass varieties with moderate sugar levels (called ‘energy cane’), sugarcane breeding programmes have been crossing commercial sugarcane varieties with *S. spontaneum* (Matsuoka *et al.*, 2014; Kumar *et al.*, 2015). Although *S. spontaneum* contributes to increased biomass, it is also early flowering, which affects yield and is an undesirable trait in commercial energy cane varieties.

As the induction of flowering causes a major developmental switch from vegetative growth to reproductive growth, it affects both sugar and biomass yields in sugarcane; this is because upon flower induction the plant stops growing and the sucrose that has been stored in the stalks is re-mobilized for use in reproductive development (Araldi *et al.*, 2010; Scortecci *et al.*, 2012). In some varieties, flowering also leads to dehydration of the stalk tissues, which negatively affects stalk density, plant weight, and also the ease of sugar extraction. Suppression of flowering through ethephon application has been shown to increase sugar yields (Li and Solomon, 2003).

Flowering in sugarcane is primarily regulated by photoperiod, temperature, age, and soil fertility. Floral induction occurs when daylength decreases below the critical photoperiod, which falls somewhere between 12.5 h and 11.5 h depending on the cultivar (Araldi *et al.*, 2010). Economic production of sugarcane in equatorial regions is hampered because flowering can occur all year round as temperatures are consistently high and the photoperiod is always close to 12 h (Clements and Awada, 1967; Scortecci *et al.*, 2012). A major goal of sugarcane breeding programmes is therefore to produce varieties that are delayed in flowering in order to increase sucrose/biomass yields and to extend the harvest season (Cheavegatti-Gianotto *et al.*, 2011; Medeiros *et al.*, 2016). Increasing sugarcane production raises many sustainability issues including deforestation and increasing land, pesticide, and fertilizer use. Delaying flowering can increase sugar/biomass yields without increasing the use of land, water, or other inputs, and is therefore an environmentally attractive approach.

Surprisingly, very little known about the genetic regulation of the flowering process in this important economic crop. A greater understanding of the control of flowering in sugarcane is needed in order to delay flowering for increased yields and seasonal extension, or to get more uniform and predictable flowering. Inconsistent flowering has impacted on breeding of new varieties as it limits the choice of desirable parents that can be used for crossing in breeding programmes (Glassop *et al.*, 2014). Some quantitative trait loci (QTLs) for flowering have been identified and there have been subtractive cDNA library studies performed on apices trying to identify genes associated with the floral transition (Ming *et al.*, 2002; Medeiros *et al.*, 2016). Recently a minimum tiling path genome sequence of sugarcane cultivar R570 has been published and is accessible via the Sugarcane Genome Hub database (Garsmeur *et al.*, 2018; NCBI genome assembly accession GCA_900465005.1).

A genome sequence assembly of a different cultivar, SP80-3280, is published in the Sucest-Fun database (NCBI genome assembly accession GCA_008692665.1; http://sucest-fun.org/). The Sucest-Fun database also contains ESTs showing homology to known flowering time genes such as *GIGANTEA* (*GI*), *CONSTANS* (*CO*), *CO-*Like, *FLOWERING LOCUS T* (*FT*), *TERMINAL FLOWER 1* (*TFL1*), and the monocot-specific genes *EARLY HEADING DATE 1* (*EHD1*) and *GRAIN NUMBER PLANT HEIGHT AND HEADING DATE 7* (*GHD7*) (Vettore *et al.*, 2003; Coelho et al., 2013, 2014). *FT* is a key floral inducer and is regulated by *GI* and *CO*; however, in rice, *Sorghum*, and other grasses, their *FT* orthologues are also up-regulated by *EHD1* and other genes that modulate *EHD1* expression such as *GHD7* (Xue *et al.*, 2008; Itoh *et al.*, 2010; Murphy *et al.*, 2014; Yang *et al.*, 2014). The identification of sugarcane homologues of *EHD1* and *GHD7* indicates that this regulatory pathway may also exist in sugarcane (Coelho *et al.*, 2013).

*FT* is just one member of a family of *FT-*like genes in plants which all encode proteins which contain phosphatidylethanolamine-binding (PEBP) domains (Wickland and Hanzawa, 2015). The *FT-*like gene family in Arabidopsis includes *TFL1*, *TWIN SISTER OF FT* (*TSF*), and *MOTHER OF FT AND TFL* (*MFT*) which promote or repress flowering (Simon *et al.*, 1996; Yoo *et al.*, 2004; Yamaguchi *et al.*, 2005). Some *FT-*like genes are, however, involved in other developmental processes such as tuberization or bulb formation rather than flowering (Navarro *et al.*, 2011; Lee *et al.*, 2013). There is a high degree of synteny between the diploid *Sorghum* and the basic sugarcane haplotype, therefore *Sorghum* is a good model for sugarcane research (Ming *et al.*, 2002; Wang *et al.*, 2010; Scortecci *et al.*, 2012). Three *Sorghum FT* orthologues have been identified that were shown to function as floral promoters in transgenic Arabidopsis *ft-1* mutant plants; these are *SbFT1*, *SbFT8*, and *SbFT10* which were previously known as *SbCN15*, *SbCN12*, and *SbCN8*, respectively (Wolabu *et al.*, 2016). Here we will use the *SbFT* terminology of Wolabu *et al.* as this maintains consistency with existing nomenclature of *FT*-like genes in sugarcane, as well as in dicots such as Arabidopsis, tobacco, etc.

Six sugarcane *FT*-like genes, *ScFT1–ScFT6*, have so far been identified and are present in the Sucest-Fun and/or NCBI databases including two (*ScFT3* and*ScFT4*) which are in the same clade as the rice *Hd3a* gene; however, which of these six *FT*-like genes are involved in floral induction in sugarcane is not known (Coelho *et al.*, 2014). Overexpression of *ScFT1* resulted in delayed flowering in Arabidopsis, suggesting that *ScFT1* is not a floral inducer (Coelho *et al.*, 2014). Glassop and Rae (2019) analysed the expression of two sugarcane *FT* genes in their diurnal RNA-seq experiments; whilst they named them *FT-A* and *FT-C*, they correspond to *ScFT3* and *ScFT1*, respectively, as described by Coelho *et al.* (2014). Here we will follow the nomenclature established in Coelho *et al.* for those genes.

We describe results of RNA-seq analyses of a number of flowering-related genes in sugarcane plants grown in either 12 h light:12 h dark, or short day (SD) 8 h light:16 h dark photoperiods, which uncovered a novel *FT*-like transcript, *ScFT12*. In total we have discovered 10 novel sugarcane PEBP genes (*ScFT7–ScFT13*, *ScMFT1*, *ScMFT2*, and *ScTFL2*) from interrogation of the Sugarcane Genome Hub database, the Sucest-Fun database, and from other RNA-seq experimental data (Vettore *et al.*, 2003; Garsmeur *et al.*, 2018). We also demonstrate flower-inducing activity of one of the sugarcane *FT* homologues (*ScFT3*) in both Arabidopsis wild-type (WT) and *ft-10* null mutant plants, indicating that this gene could be a functional *FT* homologue in sugarcane.

## Materials and methods

### Plant material for diurnal time course RNA-seq experiments

Sugarcane plants of the commercial cultivar SP83-2847 (*Saccharum* spp. hybrid), propagated by single bud stem cuttings, were grown for 60 d in a greenhouse until they reached a height of ~40 cm. They were then transferred to a growth chamber that was divided in half to provide two different photoperiodic treatments: 12 h light:12 h dark or 8 h light:16 h dark (SD), and grown for a further 30 d. Temperature in the growth chamber was maintained at a constant 27 ± 2 °C, light levels were 100 µmol m^−2^ s^−1^, and CO_2_ concentration was 405 ppm. Plants were watered twice a day and randomly reorganized weekly inside the chamber. The middle section of +1 leaves, with no vascular tissue, were harvested from six plants (12:12 photoperiod), or four plants (8:16 photoperiod), at the following time points over a period of 24 h (lights on=ZT0) as per Alves *et al.* (2019): 12h light:12h dark time course: ZT1, ZT6, ZT11, ZT13, ZT16, ZT20, and ZT23; SD 8 h light:16 h dark time course: ZT1, ZT4, ZT7, ZT9, ZT14, ZT19, and ZT23

### RNA-seq expression analysis

Total RNA was extracted using an RNeasy Plant Mini Kit (Qiagen) from 100 mg of ground leaves harvested at each time point. RNA was quantified using a NanoDrop 2000 spectrophotometer (Thermo Fisher Scientific) and quality was checked by electrophoresis. Single-end Illumina mRNA libraries were generated from 3.5 µg of total RNA using a TruSeq RNA Sample Preparation Kit (Illumina) according to the manufacturer’s protocol. Libraries were evaluated for integrity and fragment size using a Bioanalyzer (Agilent Technologies) and quantified by quantitative PCR (qPCR) with the KAPA Library Quantification for Illumina kit (KAPA Biosystems). The Illumina Hi-Seq 2500 platform was used to generate single-end 100 bp reads of the libraries.

The Illumina sequencing data were submitted to a trimming and quality checking analysis pipeline conducted with the NGS QC Toolkit (Patel and Jain, 2012). High quality reads were obtained with a minimum of 70 nucleotides at a phred score of ≥20. Transcriptome mapping was done with BowTie2 using an in-house sugarcane reference transcriptome (see below for details of how this was made), and transcript relative abundances were calculated using RSEM (Li and Dewey, 2011; Langmead and Salzberg, 2012). The read counts of unigenes from different treatments were converted into fragments per kilobase of exon model per million mapped reads (FPKM values). In order to give an accurate representation of the level of translatable mRNA in each time point sample, we clustered highly similar sequences (e.g. transcripts that contained identical protein-coding sequences) and pooled their expression values together to obtain a single representative expression value for each specific protein coding sequence.

### Sugarcane reference transcriptome

Sugarcane genotypes SP91-1049 and SP83-2847 were cultivated in the field for 9 months. Samples of +1 leaf were collected at three different times (07.00, 12.00, and 17.00 h) and RNA was extracted using the RNeasy Plant Kit (Qiagen). The RNA quality was checked by 2100 Bioanalyzer (Agilent), and single-end libraries were made using an mRNA-Seq Sample Preparation kit following the manufacturer’s instructions (Illumina Inc., San Diego, CA, USA). RNA sequencing was performed on an Illumina Hi-Seq 2500 platform. All reads were submitted to quality checking using the FASTX-Toolkit, and the read-end bases without desirable quality (q20) were trimmed. Reads that gave a Blast alignment match against yeast, bacteria, or ribosomal sequences were also excluded. In the end, a total of 630 770 178 reads were selected. The Trinity assembly pipeline was applied to create a non-redundant dataset of 44 558 361 reads (Grabherr *et al.*, 2011). These reads were assembled in different steps using the Velvet and Oases algorithms (Zerbino and Birney, 2008; Schulz *et al*., 2012). The assembly procedure was based on the concept that different sensitivities can be assessed using different k-mers. We started with k-mer 55 followed by re-assembling the transcripts using smaller k-mers (47, 39, and 31) in consecutive independent steps. The input for each step was the transcripts assembled and unused sequences from the last assembly. This approach results in 191 871 sugarcane transcript sequences (available in the Dryad Data Repository https://doi.org.10.5061/dryad.fn2z34tv2) (Venail *et al*., 2022).

### Annotation of RNA-seq transcripts

To identify sugarcane homologues of known flowering-related genes, Blastx searches using the sugarcane transcriptome as queries were performed against a bait dataset of protein sequences from *Arabidopsis thaliana*, *Sorghum bicolor*, and *Oryza sativa*. The sugarcane coding sequences were deduced from the amino acid alignment with the blastx best-match (Vicentini *et al.*, 2012). The translated sugarcane sequences were then aligned with the 40 first blastx hits by MAFFT using default parameters (Katoh *et al.*, 2005). The phylogenetic relationship of the aligned protein sequences was then inferred by maximum likelihood (ML) using PhyML with the WAG plus gamma substitution model and aLTR test (Anisimova and Gascuel, 2006; Guindon *et al.*, 2010).

### Gene tree analysis

Sequences from the RNA-seq data identified as members of the PEBP gene family were used to query the Sucest-Fun (http://sucest-fun.org/), the Sugarcane Genome Hub (https://sugarcane-genome.cirad.fr), and GenBank (https://www.ncbi.nlm.nih.gov/genbank/) databases (Vettore *et al.*, 2003; Garsmeur *et al.*, 2018). This identified previously published sugarcane *FT*-like sequences (*ScFT1–ScFT6*), as well as *TFL*-like (*ScTFL1*, *3*, and *4*), and *MFT*-like (*ScMFT1*) sequences (as shown in Supplementary Table S1A). Novel *FT*-like (*ScFT7–ScFT13*), *TFL-*like (*ScTFL2*), and *MFT-*like (*ScMFT1* and *ScMFT2*) sequences were also identified and, whenever possible, assigned names to match their *Sorghum* homologues according to the nomenclature of Wolabu *et al.*, (2016). The 19 sugarcane PEBP genes identified were compared with homologous genes from *Sorghum*, rice, maize, and Arabidopsis to create the gene trees (gene reference numbers are shown in Supplementary Tables S1A, B).

Protein sequences translated from gene coding regions were used for the alignment due to the low level of DNA sequence homology over the whole group. Amino acid sequences were initially aligned using ClustalW (http://www.clustal.org), and the alignments subsequently manually refined in Mesquite (http://www.mesquiteproject.org;Maddison and Maddison, 2019). Topologies were then searched and assessed via PAUP v4.0 b167 using built-in implementations of optimality criteria (Swofford, 2001). The tree was generated by heuristic search of a distance matrix under the balanced minimum evolution criterion. The general matrix was composed of 178 amino acids covering the nearly complete protein sequence (N- and C-termini were trimmed for certain taxa to make the alignment a coherent block). Bootstrap support values were obtained with 10 000 replicates of a heuristic search under the same optimality criterion. Identical topologies were recovered either by using other optimality criteria (Fitch–Margoliash weighted least square), or by parsimony and ML methods. The topologies were reimported into Mesquite for graphical representation.

The tree of the *TFL* genes presented in Supplementary Fig. S1was obtained by aligning the full-length nucleotide coding sequences from sequences available in Sucest-Fun and the Sugarcane Genome Hub. Several contigs were found for each of the *TFL*-like homologues, but sequences already published were used as references when available (*ScTFL1*, *ScTFL3*, and *ScTFL4*). Of the seven contigs in the Sucest-Fun database found to contain *ScTFL2*, four had identical sequences, and this was used as the reference sequence for the alignment. All genomic sequences from sugarcane and *Sorghum* were aligned as described above, and their intron–exon borders defined. All coding sequences were checked to be in-frame and matching previously published protein sequences. Subsequently a gene tree based on their cDNAs was constructed using an ML analysis, with a K80+G model (AICc=4206.591), equal base frequencies, a gamma distribution with a shape of 0.39, and a Ti/Tv ratio of 1.92 as suggested by Modeltest v3.7 (Posada and Crandall, 1998).

### Flowering analysis in Arabidopsis

The full-length coding sequence for *ScFT3* was obtained from our RNA-seq data and the full-length *ScFT5* sequence was obtained from the Sucest-Fun database (Vettore *et al.*, 2003). The complete coding sequences of *ScFT3* and *ScFT5* were synthesized by Integrated DNA Technologies (IDT). Due to the high GC content of the 5ʹ end of the *ScFT3* sequence, it had to be altered according to their recommendations to enable synthesis; however, only synonymous substitutions were made which did not affect the protein sequence of the ScFT3 protein (Supplementary Fig. S2). The synthesized fragments were introduced by Gateway recombination in pB7WG2 under the control of the 35S promoter (Karimi *et al.*, 2002). The recombinant plasmids were then transformed in *Agrobacterium* GV3101.

Arabidopsis WT and *ft-10* mutant (line GK-290E08 from NASC, Scholl *et al.*, 2000; Yoo *et al.*, 2005) plants were transformed using the floral dip method with constructs directing the overexpression of full-length *ScFT3* and *ScFT5*. As controls, constructs where the ATG start codons of the *ScFT3* and *ScFT5* genes had been mutated to disrupt translation of the full-length proteins (∗*ScFT3* and ∗*ScFT5*) were also created and transformed into WT and *ft-10* mutant Arabidopsis plants. Between 16 and 28 BASTA-resistant T_1_ transformed lines from each transformation were grown in growth cabinets in SD conditions at a constant 22 °C, and the rosette leaf number at flowering was recorded. Plants were checked for insertion of the transgene by PCR and sequencing of the PCR product. Any plants that still had not flowered after forming 10 leaves were then transferred to long days (LDs) to induce flowering to obtain T_2_ seed. Fifty T_2_ seed from each line were germinated on agar containing BASTA to identify lines with single T-DNA insertions (3:1 ratio of resistant:sensitive). For each construct, eight plants from three independent BASTA-resistant single insertion T_2_ lines were then transferred to soil and grown on in SD conditions alongside non-transformed WT and *ft-10* mutant plants in order to record the rosette leaf number at flowering.

### Developmental time course expression analysis of *ScFT3*

The expression of *ScFT3* was investigated over a developmental time course in the sugarcane cultivar IACSP96-7569 at seven specific time points covering a period of 13 weeks designed to span the initial point of floral induction in SD-grown plants, usually occurring within 6 weeks, plus a couple of later time points several weeks post-induction (samples were taken from three biological replicates in both SD and LD treatments, at the same time of day in weeks 2, 3, 4, 5, 6, 9, and 13 following the start of the SD photoperiodic treatment). This experiment was conducted in an automated photoperiod facility at the IAC Centro de Cana (Ribeirão Preto, São Paulo, Brazil). Plants were grown from single bud stem cuttings and grown for 7 months in LD conditions. The plants were then divided between two trolleys that could move in and out of two photoperiod chambers (Supplementary Fig. S3; Melloni *et al.*, 2015; Manechini *et al.*, 2021) and could thus be subjected to two different photoperiod treatments: either a constant LD photoperiod of 13 h and 30 min, or an SD photoperiod of 12 h and 50 min shortened by 45 s per day. The plants maintained on the trolleys were grown as much as possible outside the chambers in natural sunlight (equal for both LD and SD treatments) and then pulled into the chamber to complete the rest of the photoperiod treatment. For each sampling time point, the shoot apical meristem (SAM) and the mature leaf +1 (the first fully expanded leaf that has a visible dewlap which is a band of membranous tissue between the leaf sheath and the leaf blade) were collected in the late afternoon from three different plants in both photoperiodic treatments. The leaf was immediately frozen in liquid nitrogen and stored at –80 °C while the SAM was fixed in FAA 50% (formalin, acetic acid, and ethyl alcohol) solution for histological sectioning using a microtome to verify the developmental stage of the SAM. The SAM of plants grown for 13 weeks in SD inducing conditions was sufficiently large to visualize without sectioning and staining.

Total RNA was extracted using a PureLink RNA Mini Kit (ThermoFisher) and cDNA synthesis using a QuantiNova Reverse Transcription kit (QIAGEN) following the manufacturer’s instructions. Real-time PCR was conducted using a Bio-Rad IQ5 machine. The *ScFT3* primers used were: forward 5ʹ-TGTCTACTTCAACGGCCAAA-3ʹ, and reverse 5ʹ-CCCAACTACGTACCCATCATC-3ʹ. Published primers for the *ScUBQ*1 and *ScTUB* genes (de Andrade *et al.*, 2017; da Silva Santos *et al.*, 2021) were used to normalize the *ScFT3* expression data. Amplification conditions were: 95 °C for 3 min, followed by 40 cycles of 10 s at 95 °C and 30 s at 60 °C, followed by a melting curve from 55 °C to 95 °C. Relative expression data and statistical analysis were performed using the software REST 2009, with 2000 interactions and differences considered significant when *P*<0.01 (Pfaffl *et al.*, 2002). The inductive SD treatment was considered ‘treated’ and the non-inductive LD treatment was considered ‘untreated’ or control for the purpose of the software expression calculations.

## Results

### Sugarcane diurnal transcriptome analysis

**Fig. 1.**
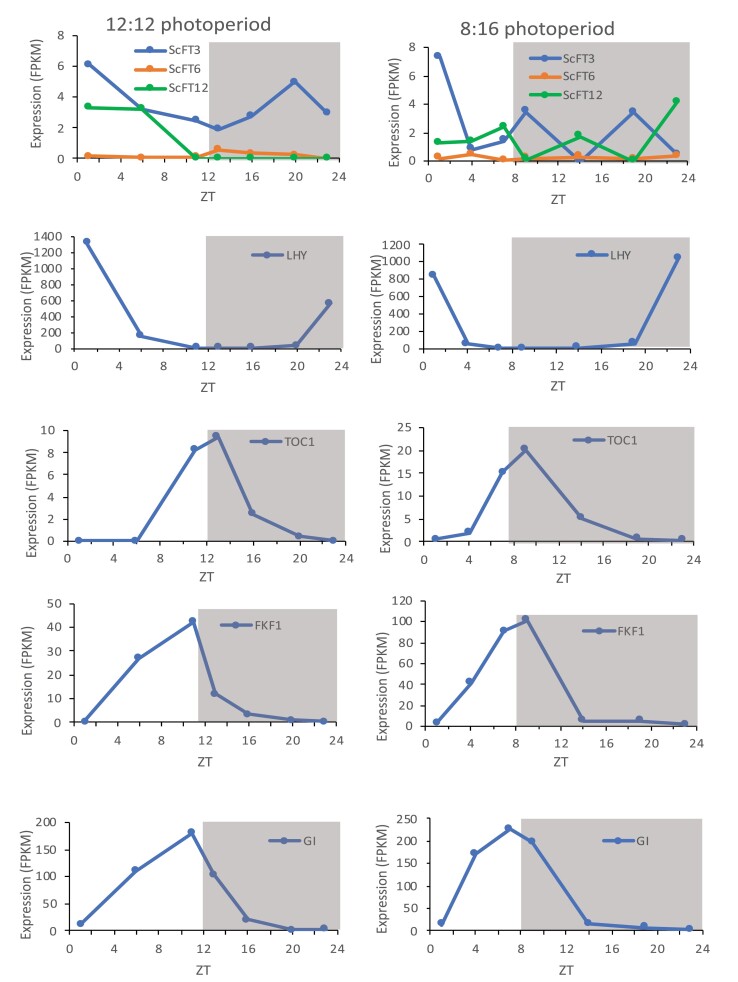
Diurnal expression of sugarcane flowering- and circadian-related genes in sugarcane plants grown in either a 12 h light:12 h dark photoperiod (left), or a short day 8 h light:16 h dark photoperiod (right). ZT is time (hours) from lights on. The read counts of unigenes from different treatments were converted into fragments per kilobase of exon model per million mapped reads (FPKM values) using the RNA-Seq by Expectation Maximization software package (Li and Dewey, 2011).

RNA-seq diurnal time course experiments aiming to identify sugarcane genes whose expression in leaves cycled throughout the day were conducted on young plants grown in both 12 h:12 h and SD 8 h light:16 h dark photoperiods (Alves *et al.*, 2019). The expression of three sugarcane *FT*-like transcripts in leaves was detected by these RNA-seq analyses (Fig. 1). One *FT*-like transcript encoding a predicted full-length ScFT3 protein showed high expression at the beginning of the day and subsequent peak(s) in the dark period. The diurnal expression pattern of the *ScFT3* transcript in our conditions (high at the start of the day, then low for the rest of the light period before increasing again in the night) is similar to the published expression pattern of *Hd3a* in rice, another SD plant (Kojima *et al.*, 2002; Hayama *et al.*, 2003). It is also consistent with the expression pattern reported by Glassop and Rae (2019) for the same gene (called *FT-A* in that paper) even though the photoperiod they used was slightly different (14 h light:10 h dark); whilst this is likely to be a non-inductive photoperiod, *FT* genes (including the Arabidopsis *FT* and rice *Hd3a*) are known to also be expressed in non-inducing conditions albeit at a lower level (Kardailsky *et al.*, 1999; Hayama *et al.*, 2003).

The other two *FT*-like transcripts detected in our diurnal RNA-seq experiments were expressed at lower levels than *ScFT3* (Fig. 1). One of these transcripts was *ScFT6* and was expressed at very low levels in both photoperiods. The other transcript (transcript_118650) had no homology to any of the six *ScFT* genes present in the Sucest-Fun or GenBank databases, and turned out to be a partial, or mis-spliced (or mis-assembled during RNA-seq assembly), transcript of a novel sugarcane *FT*-like gene (*ScFT12*; Supplementary Fig. S4). A further transcript with homology to *ScFT2* (transcript_110985; Supplementary Fig. S5), was also identified at very low levels in just one sample (ZT19) of the 8:16 SD diurnal time course data; the expression level was too low to show in Fig. 1. Other *FT*-like genes may have been expressed at levels below detection in our experimental conditions.

The expression of other flowering- and circadian-related genes (Fig. 1: *LHY*, *TOC1*, *FKF1*, and *GI*) in our experimental conditions showed diurnal expression patterns consistent and comparable with those observed in other species for those genes in SD and LD photoperiods including, for example, the shift in the peak expression of *GI* to earlier in the day in shorter photoperiods compared with longer photoperiods (Fowler *et al.*, 1999; Alabadi *et al.*, 2001; Sawa *et al.*, 2007).

### Identification of other members of the PEBP family in sugarcane

A comparison of the sugarcane FT-like protein-coding sequences identified from all our different RNA-seq analyses was made with FT-related proteins from other species (Supplementary Table S1B). This, together with mining of the Sucest-Fun database and the Sugarcane Genome Hub, enabled the identification of another seven *FT*-like gene sequences (*ScFT7*–*ScFT13*), two *MFT*-like genes (*ScMFT1* and *ScMFT2*), and one *TFL*-like gene (*ScTFL2*) from sugarcane, all having corresponding homologues in other monocot species such as *Sorghum*, maize, and rice.

**Fig. 2.**
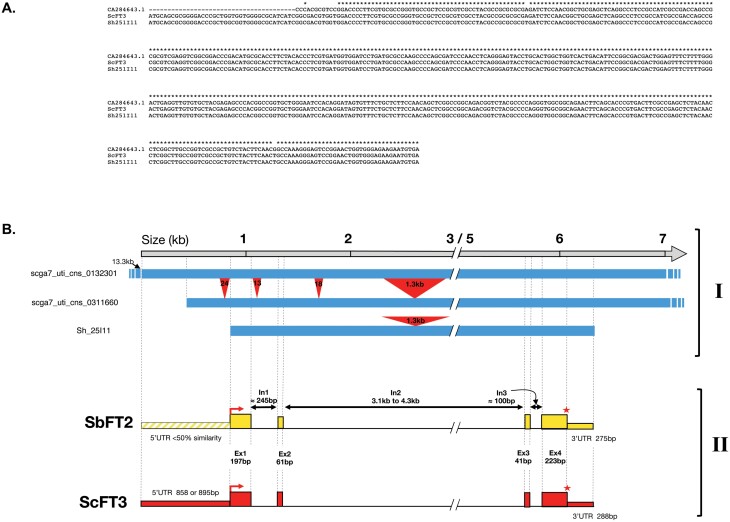
Sequence and structure of the sugarcane *ScFT3* gene. (A). Alignment of ScFT3 coding sequences; CA284643 from Coelho *et al.* (2014); *ScFT3* RNA-seq transcript from this study; Sh251I11 from the Sugarcane Genome Hub database. (B) I: alignment of contigs containing the predicted transcript sequence. The top grey line indicates the distances relative to the consensus sequence of the alignment (including insertions). The blue lines indicate matching genomic sequence (>90% similarity), with the black arrow indicating the point of alignment of the top contig. Indels (>5 bp) are represented by red rectangles with the pointed end marking deletion in one contig and the opposite side corresponding to an insertion in the other. The introduced gap represents a large non-coding intronic region that has not been included for graphical representation. BAC ‘Sh_251I11’ from the Sugarcane Genome Hub database contains the predicted complete full-length transcript (Sh_251I11p000030). Two contigs from the Sucest-Fun database are represented: ‘scga7_uti_cns_0132301’ which covers the full length of the transcript, and ‘scga7_uti_cns_0311660’, which lacks the 5ʹ end of the 5ʹ-UTR (see Supplementary Table S2). II: gene structure of the sugarcane *ScFT3* gene aligned to its closest *Sorghum* homologue (*SbFT2*) and the sugarcane contigs. The red arrows correspond to translational starts, the red stars to stop codons.

A full-length transcript sequence of *ScFT3* was obtained from our diurnal RNA-seq experiments which extended the coding region of the existing *ScFT3* cDNA sequence in the Sucest-Fun and GenBank databases (CA284643.1) by 50 bp in the 5ʹ direction to the start codon (Fig. 2A). This was confirmed by the subsequent identification of a predicted full-length *ScFT3* transcript sequence Sh_251I11_t000030 in the Sugarcane Genome Hub BAC Sh_251I11 (Fig. 2B). The closest homologue from *Sorghum* is *SbFT2* which shares a coding sequence similarity of 97% and a conserved intron–exon structure with *ScFT3*. The 5ʹ-untranslated regions (UTRs) have <50% sequence similarity but there is 89% similarity in the 3ʹ-UTRs of the two genes.

**Fig 3.**
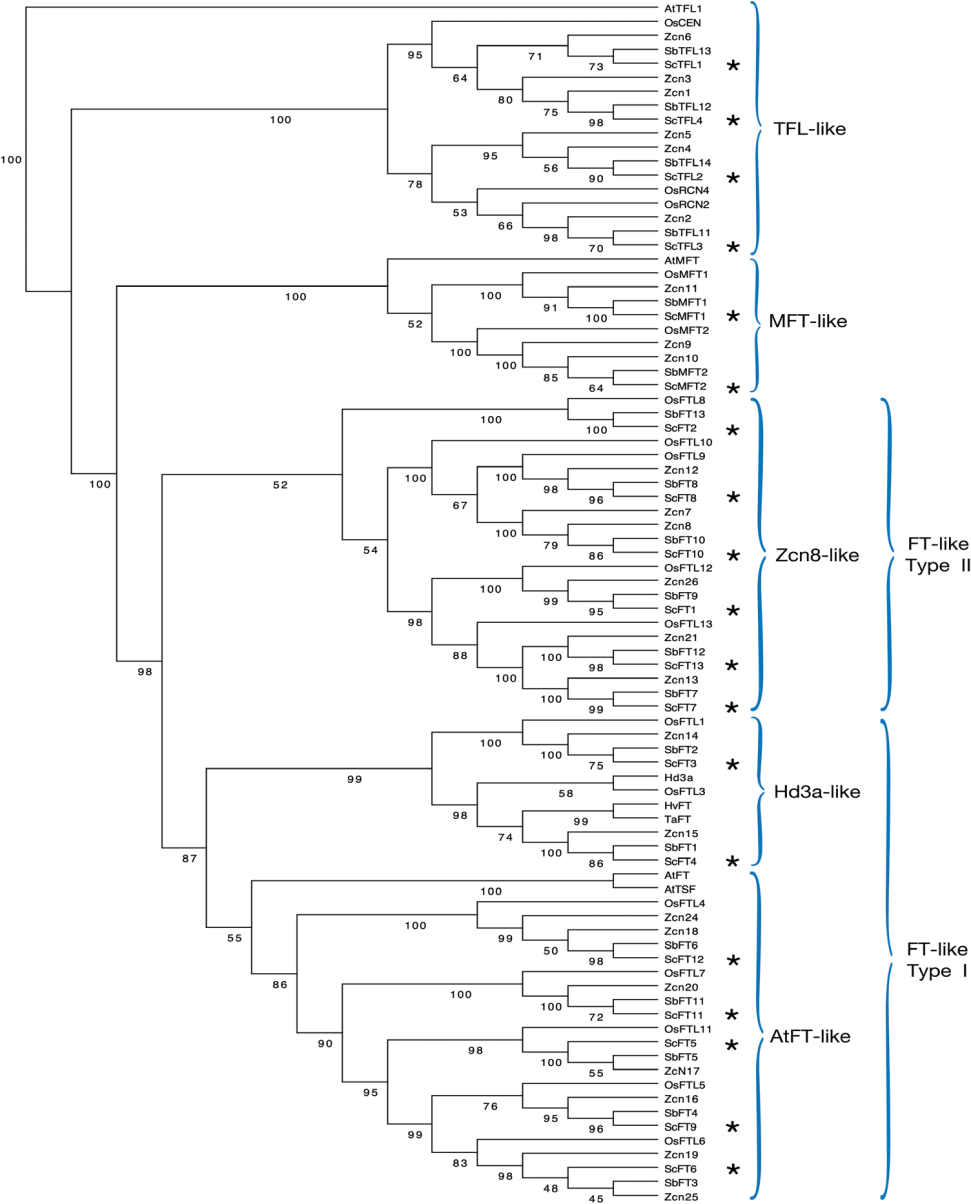
Gene tree of FT/PEPB-related proteins in sugarcane and other species. This topology is based on protein sequences of PEBP genes in sugarcane, maize, and *Sorghum*. Sugarcane sequences are indicated with asterisks. Boostrap values (10 000 replicates) are indicated above the branches. *AtTFL1* was set as an outgroup for graphical representation. Species abbreviations: *ScFT*, sugarcane; S*bFT*, Sorghum; *Zcn*, maize; *Hd3a*, *OsCEN*, *OsFTL*, rice; *TaFT*, wheat; *HvFT*, barley; At, Arabidopsis. References for the sugarcane protein sequences used are presented in Supplementary Table S1A, and for those of other species in Supplementary Table S1B.

The deduced intron–exon structures of the PEBP genes shown in the gene structure figures (Fig. 2B; Supplementary Figs S4–S20) were determined by alignment of the cDNA sequences to their genomic counterparts. Most *FT*-like, *MFT-*like, and *TFL*-like genes show the conserved structure of four exons that is generally observed in the PEBP gene family, and usually exons 2 and 3 are conserved in size (61–63 bp and 40–42 bp, respectively), consistent with other plant PEBPs (Carmona *et al.*, 2007; Danilevskaya *et al.*, 2008). In sugarcane, there are a few exceptions to this canonical arrangement. Firstly, *ScFT11* has just two exons and a single intron as a result of the fusion of exons 1 and 2, and 3 and 4, and this is also seen in its *Sorghum* homologue *SbFT11* (Supplementary Fig. S14). As shown in Fig. 3, *ScFT11* and *SbFT11* are in the same subclade as *ZCN20* in which only the first two exons have fused and just the first intron has been lost (Danilevskaya *et al.*, 2008). A second exception is *ScTFL3* for which the first intron has been lost, and exons 1 and 2 are fused (Supplementary Fig. S19). This loss of the first intron is also observed in its homologues in *Sorghum* and maize, *SbTFL1-1* and *ZCN2*, respectively. A third variant in intron number is *ScFT12* which contains four introns. Its homologue in *Sorghum* (*SbFT6*) has previously been described by Wolabu *et al.* (2016) as containing three introns, but upon careful examination of different genomic sequences, and alignment to expressed transcripts (transcript_118650 from this study, *SbFT6* transcript described in Wolabu *et al.*, 2016, and the predicted transcript Sh_210B16_p000100 from Garsmeur *et al.*, 2018), a fourth intron of ~600 bp was shown to be present splitting the fourth exon in two (Supplementary Fig. S4). The observed variation in intron–exon structure of homologous members of the PEBP gene family in sugarcane, *Sorghum*, and maize (ZCN20, SbFT11, and ScFT11; ScTFL3, SbTFL1-1, and ZCN2; and ScFT12 and SbFT6) may be evidence of recurrent evolutionary loss of specific introns, a process which is not uncommon (Wang *et al.*, 2014).

### Phylogenetic analysis of sugarcane FT homologues

To investigate the relationship between the existing and newly identified *FT*-like genes, and also the relationships to homologues from other species, we produced a PEBP gene family tree (Fig. 3). The topology obtained shows a clear clustering of clades in the gene tree concordant with previously published data in sugarcane, *Sorghum*, and maize (Danilevskaya *et al.*, 2008; Coelho et al., 2013, 2014; Wolabu *et al.*, 2016). Homologous genes from each of these species cluster in the same clade and the distinction between families of *TFL1*-like, *MFT*-like, and *FT*-like genes is maintained. In the *TFL*-like clade, all four *ScTFL* genes are grouped closely with their *Sorghum* homologues (Fig. 3; Supplementary Fig. S1). The *MFT*-like clade is well defined and includes *ScMFT1* and *ScMTF2* which both have high homology to the *Sorghum SbMFT1* and *SbMFT2* genes. The close pairing of the homologous sugarcane and *Sorghum FT-*like genes illustrates the low level of divergence between the respective PEBP gene sequences in these two closely related species.

The *FT*-like clade can be subdivided into Type I and Type II as per Danilevskaya *et al.* (2008), and the *FT*-like I clade can be further subdivided into two subclades, one containing *AtFT*, and a second *Hd3a*-like subclade. In this second *Hd3a*-like subclade, *ScFT3* is grouped with *SbFT2* which has been shown not to be able to trigger flowering when overexpressed in Arabidopsis, and their closest homologue *ZCN14* is also unlikely to be a floral activator considering that it is expressed in tassels and kernels (Danilevskaya *et al.*, 2008; Wolabu *et al.*, 2016). The other group within the *Hd3a*-like clade includes *ZCN15*, *ScFT4*, *SbFT1*, and *Hd3a* itself. Both *Hd3a* and *SbFT1* have been shown to be floral activators; however, *ZCN15* accumulates in maize kernels after fertilization and is therefore unlikely to be involved in floral induction. The *AtFT*-like subclade of *FT-*like I genes also contains genes that are able to induce flowering and genes that cannot: *ZCN18* did not induce early flowering in transgenic maize, and the closely related *SbFT6* does not complement the *ft-1* mutation in Arabidopsis (Meng *et al.*, 2011; Wolabu *et al.*, 2016). Thus, whilst it is likely that homologues grouped in the same clade probably share some commmon ancestry, some are floral activators whereas others cannot induce flowering.

The *FT*-like II clade contains genes unique to monocots, including *ZCN8* from maize which has been shown to have floral inducing activity in Arabidopsis (Lazakis *et al.*, 2011). *ZCN8* clusters with *ScFT10* and *SbFT10*; the latter has also been shown to induce flowering in the *ft-1* mutant in Arabidopsis, but in addition induces major pleiotropic effects when overexpressed. *ScFT8* is grouped with *SbFT8* which is also able to induce flowering in the Arabidopsis *ft-1* mutant whilst at the same time causing some pleiotropic effects (Wolabu *et al.*, 2016). It remains to be tested whether the sugarcane *ScFT8* and *ScFT10* genes could also be floral activators like their homologues in *Sorghum*. *ScFT1* has been shown to be a repressor of floral transition when overexpressed in Arabidopsis, and it is grouped with *SbFT9* and *ZCN26* which also do not exhibit any floral inductive activity (Meng *et al.*, 2011; Coelho *et al.*, 2014; Wolabu *et al.*, 2016).

Some of the paired genes from sugarcane and *Sorghum* could represent orthologous genes, but this is difficult to prove without a functional test because of the polyploid origin of those species. So despite *ScFT3* being paired with *SbFT2* (which is not able to induce flowering), its expression profile is similar to that of the rice *Hd3a* gene which is another close homologue and is a floral activator (Kojima *et al.*, 2002; Hayama *et al.*, 2003). For this reason, we decided to test *ScFT3* together with *ScFT5* (a close homologue to Arabidopsis *FT*) for their ability to induce flowering in Arabidopsis.

### Flowering analysis in transgenic Arabidopsis

To assess the ability of sugarcane *FT*-like genes to induce flowering, transgenic studies in Arabidopsis were performed. It has previously been shown that overexpression of *ScFT1* in Arabidopsis did not cause early flowering, and in fact delayed flowering, suggesting that *ScFT1* may not be the functional *FT* orthologue in sugarcane (Coelho *et al.*, 2014). Gene tree analysis (Fig. 3) shows that *ScFT3* and *ScFT4* are the closest sugarcane homologues to rice *Hd3a*. However, only *ScFT3* was taken forward to test for its flower-inducing ability in Arabidopsis as we did not detect expression of *ScFT4* in our RNA-seq experiments. In addition, when we started this work, *ScFT5* from the *AtFT*-like subclade of *FT-*like I genes was the closest known sugarcane homologue to the Arabidopsis *FT* gene, so it was therefore also tested for possible flower-inducing capability in the Arabidopsis *ft-10* mutant.

Arabidopsis WT and *ft-10* mutant plants were transformed with constructs directing the overexpression of full-length ScFT3 and ScFT5 proteins. The full-length coding sequence for *ScFT3* was derived from our RNA-seq experiments as we have shown that the GenBank entry at the time (CA284643.1) lacked the 5ʹ end of the coding sequence (Fig. 2A). The full-length *ScFT5* sequence was obtained from the Sucest-Fun database (Vettore *et al.*, 2003). In addition to non-transformed WT and *ft-10* mutant Arabidopsis plants, negative controls with mutated ATG start codons for both *ScFT3* (*∗ScFT3*) and *ScFT5* (∗*ScFT5*) were generated and transformed into WT and *ft-10* mutant Arabidopsis plants. Between 16 and 28 BASTA-resistant T1 plants were obtained for each of the transformed lines (*ScFT3*, ∗*ScFT3*, *ScFT5*, and ∗*ScFT5*).

**Fig. 4.**
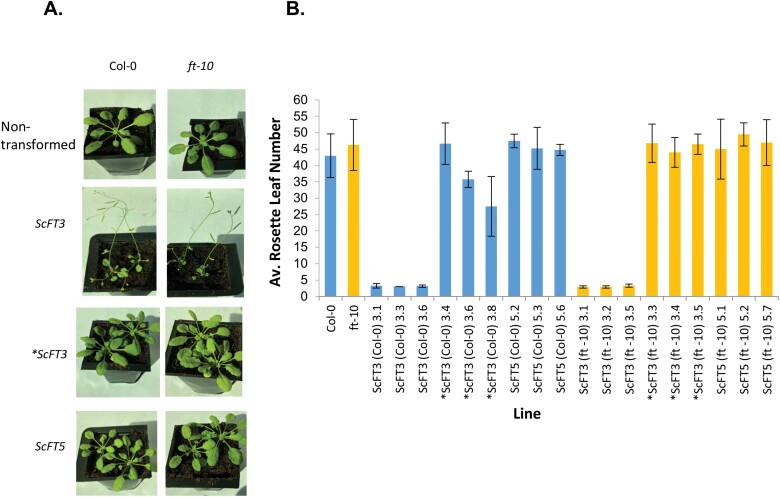
(A) Photos of representative T_2_ transgenic Arabidopsis lines, together with non-transformed WT Col-0 and *ft-10* mutant control plants, grown in 8 h SDs. Photos of all plants were taken at the same time 6 weeks after planting. WT and *ft-10* plants overexpressing the *ScFT3* transgene flowered much earlier in SD conditions than either non-transformed plants or plants overexpressing the mutated *∗ScFT3* or *ScFT5* transgenes. (B) Average rosette leaf number at flowering of T_2_ Arabidopsis plants transformed with functional *ScFT-3*, *ScFT-5*, or non-functional *∗ScFT-3* in either Col-0 (blue) or *ft-10* mutant (orange) backgrounds. Error bars show the standard deviation.

Eight plants from three independent single insertion T_2_ lines per construct were grown in SD conditions alongside WT and *ft-10* mutant plants, and the rosette leaf number at flowering was recorded. As shown in Fig. 4, all of the T_2_ single insertion lines transformed with functional *ScFT3* in either Col-0 or *ft-10* mutant backgrounds flowered very early in SD conditions (after forming between three and four rosette leaves), much earlier than plants transformed with the mutated non-functional *ScFT3* (*∗ScFT3*) or plants overexpressing *ScFT5* or *∗ScFT5* (data not shown), which both flowered much later with >27 leaves similar to the non-transformed WT and *ft-10* plants. In all other respects, the T_2_ overexpressing lines looked exactly like WT plants and had no visible growth or developmental impairments. In summary, only the functional *ScFT3* gene was able to induce early flowering in WT and *ft-10* mutant plants, whereas the functional *ScFT5* gene and the non-functional gene constructs (*∗ScFT3* and *∗ScFT5*) had no effect on flowering time.

### Developmental time course expression of ScFT3

**Fig. 5.**
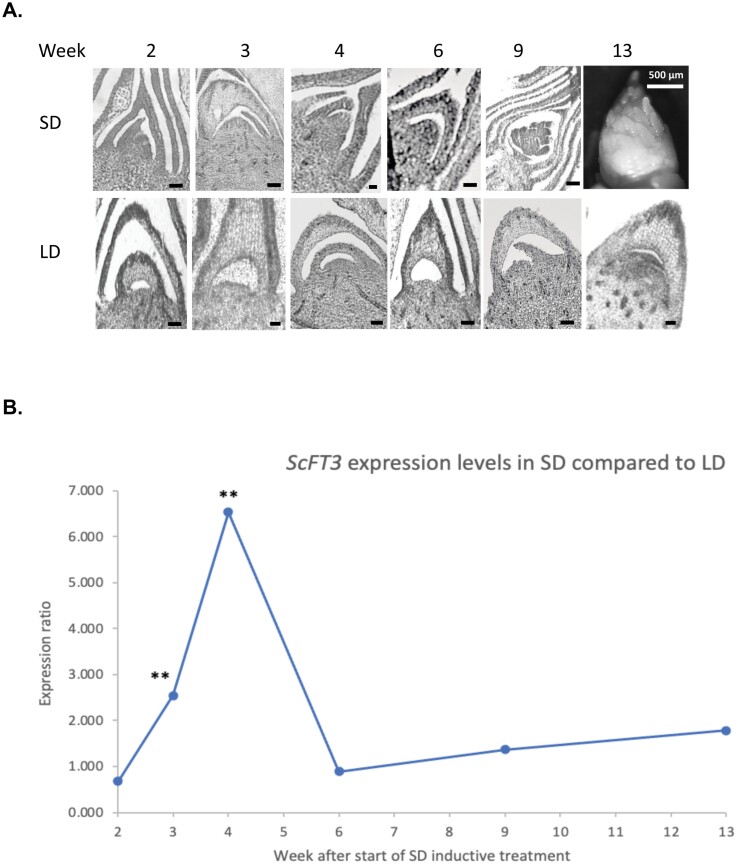
A. Apical meristem dissections of sugarcane plants grown in SDs over a period of 13 weeks from the start of inductive SD treatments (top) compared with plants maintained in LDs for the whole of that period (bottom). Black scale bars=100 µm. The apical meristem of the SD 13-week-old plant was large enough to be visible under the stereomicroscope and did not need to be sectioned. (B) Average relative expression (SD/LD) of *ScFT3* in mature leaf samples from three independent sugarcane plants harvested over the 13 week time course from the start of inductive SD treatments. Values were calculated using REST. Significant differences between average expression levels in SD and LD are indicated ∗∗*P*<0.01 (see Supplementary Table S3 for values).

The expression of *ScFT3* in mature leaves of sugarcane plants grown in inducing SD and non-inducing LD photoperiods was examined by reverse transcription–qPCR (RT–qPCR) over a 13 week period spanning the period of floral induction in plants grown in inducing SD photoperiods. Seven-month-old mature sugarcane plants exhibiting up to nine mature internodes were used as the starting material because plants showing 7–8 fully expanded internodes are considered sufficiently mature to respond well to photoperiod stimulus (Glassop *et al.*, 2014). The photoperiod regime for the SD treatment (12 h and 50 min shortened by 45 s per day) was evidently effective in promoting flowering as flag leaves and inflorescences emerged in the majority of plants in the SD treatment (Supplementary Fig. S3), but were absent in all of the plants in the LD treatment. Apical meristem samples were collected at each sampling time point and dissected to determine when floral induction had initiated (Fig. 5A). The histological sections observed under a light microscope show that, compared with the SAMs of LD plants, the transition of the SAM to an elongated dome shape appears to be starting around the third or fourth week SD time point, with the SAM being clearly elongated by the fourth week after the start of the SD inductive photoperiod. The development of inflorescence primordia is clearly visible at the ninth and 13th week SD time points, whereas for the plants grown in LD no elongation of the SAM or development of inflorescence primordia was observed throughout the duration of this experiment.

The RT–qPCR analysis showed that *ScFT3* expression is dramatically up-regulated in mature leaves 4 weeks after the start of the SD inductive treatment compared with LD (Fig. 5B). The individual SD and LD expression levels relative to two reference genes (Sc*TUB* and *ScUBQ1*) are shown in Supplementary Fig. S21. The expression of *ScFT3* in LDs can be seen to be very low in weeks 3 and 4 when floral induction occurs in SDs (as measured by elongation of the SAM). Due to poor quality RNA, the fifth week samples are unfortunately missing from these expression analyses. The subsequent increase in *FT* expression in older plants observed at 9 and 13 weeks (2 or 3 months after the start of the experiment) in both SD and LD conditions is likely to be due to the de-repression of *FT* expression by the age-related pathway (Wang, 2014).

## Discussion

### The PEBP gene family in sugarcane

The growing availability of genomes from different sugarcane cultivars allowed us to perform an exhaustive search of PEBP genes in sugarcane (Riaño-Pachón and Mattiello, 2017; Garsmeur *et al.*, 2018; Souza *et al.*, 2019). Sequence analogy of the conserved motifs specific to PEBP gene family members enabled us to identify seven new putative *FT*-like genes, two *MFT*-like genes, and a *TFL*-like gene, all with matching counterparts in maize and *Sorghum* (Supplementary Figs S4-S20; Supplementary Tables S1A, 1B). This brings the number of PEBP genes in sugarcane to 19, the same as in its closest relative *Sorghum*.

Our phylogenetic analysis also reveals that the newly described *ScFT* genes cluster in the same subclades as related homologues from maize and *Sorghum* (Fig. 3). No assumptions can be made about their function purely based on their similarity to their homologues in maize and *Sorghum* as subclades can include both functional and non-functional *FT-*like genes, such as *ScFT3* and *SbFT2*. This is something that has also been seen in other species such as tobacco, onion, and *Beta vulgaris*, which have all been shown to have *FT*-like genes that act as either repressors or activators (Pin *et al.*, 2010; Harig *et al.*, 2012; Lee *et al.*, 2013). Each *FT-*like gene will therefore have to be assessed independently to fully understand its role in the control of flowering in sugarcane.

### Identification of an ScFT orthologue

As we have shown that there are at least 13 *FT*-like genes in sugarcane (excluding the *MFT* and *TFL* homologues), we wanted to determine which of these *FT*-like genes are involved in floral induction in sugarcane and thus are true *FT* orthologues. A well-established approach to determine gene function is through functional studies in an Arabidopsis mutant deficient in the activity of the equivalent gene. In our case, we chose to examine whether either *ScFT3* or *ScFT5* was capable of rescuing the late flowering phenotype of the Arabidopsis *ft-10* late flowering mutant. As the diurnal expression pattern of *ScFT3* was similar to that of *OsHd3a*, its close homologue in rice, it was a good candidate to be tested. We also tested *ScFT5* for its ability to complement the Arabidopsis *ft-10* mutant because when we started this work it was the closest known sugarcane homologue to the Arabidopsis *FT* gene (we now know that *ScFT6*, *ScFT9*, *ScFT11*, and *ScFT12* are also in the same clade and their ability to complement the Arabidopsis *ft-10* mutant will also need to be tested).

WT and *ft-10* mutant Arabidopsis plants overexpressing *ScFT3* were very early flowering in SD conditions compared with transformants overexpressing the mutated version of this gene (∗*ScFT3*) which flowered at the same time as non-transformed WT and *ft-10* mutant plants, and transformed *ScFT5* plants. This was observed in both individual T_1_ transformed plants (data not shown), and in three independent T_2_ lines that contained just single T-DNA insertions (Fig. 4). This demonstrates that the ScFT3 protein is able to induce early flowering and is thus a floral inducer, whereas ScFT5 is not.

The closest homologue to *ScFT3* in *Sorghum* is *SbFT2*, which has been demonstrated not to complement the *FT* function in Arabidopsis (Wolabu *et al.*, 2016). The protein sequences of ScFT3 and SbFT2 are identical except for a single amino acid change at position 120 (an isoleucine in ScFT3, and a methionine in SbFT2). As this position is one of the three which have been shown to be crucial for the function of PEBP genes in Arabidopsis, the methionine at this position in SbFT2 could explain why it is unable to trigger floral transition when overexpressed in Arabidopsis, whereas the ScFT3 protein with an isoleucine at this position is able to (Hanzawa *et al.*, 2005; Wolabu *et al.*, 2016). Considering that sugarcane and *Sorghum* are regarded as close relatives, this illustrates the rapid evolution of this gene family. Single mutations leading to large phenotypic changes and potential rapid radiation have been shown to be a widespread phenomenon in both plants and animals; for example, a single mutation is thought to be behind an adaptive shift in pollinator preference, from bumblebees to hummingbirds, in Monkeyflower (*Mimulus*) (Bradshaw and Schemske, 2003). Industrial melanism in the peppered moth (i.e. the evolution of its colour from pale to black providing it with better camouflage in an industrial environment) is due to a single mutational event that arose in 1819 (Van’t Hof *et al.*, 2016).

### Developmental time course expression of *ScFT3*

Higher levels of *FT* gene expression in the leaves of induced plants would be expected to precede visible changes in the SAM as the FT protein that is made in the leaves has to move from the leaves to the apex to induce the floral transition. In sugarcane we detected a 2.5-fold up-regulation of Sc*FT3* expression in mature leaves of SD plants compared with LD plants at the third week after the start of the SD inductive treatment. By the fourth week in SDs, this had increased to almost a 7-fold induction, with elongation of the apical dome (signalling the change from vegetative to reproductive meristem development) appearing to start around this time (Fig. 5). As the inductive treatment involved reducing the photoperiod by 45 s per day from a starting daylength of 12 h 50 min, we do not know at exactly what point (i.e. which day) in the experiment the decreasing photoperiod was perceived as inductive by our sugarcane cultivar; however, as increased levels of expression of *ScFT3* were detected 3 weeks from the start of the SD treatment, it suggests that the photoperiod became inductive around that time point (with daylength being shortened by 45 s d^–1^, a 3 week period would equate to a reduction of 16 min from the initial photoperiod of 1 2h 50 min, meaning that the photoperiod probably became inductive at a daylength of ~12 h 35 min for our sugarcane cultivar IACSP96-7569).

Although all the plants used in the experiment represent the same genotype that had been vegetatively propagated by bud chips, developmental differences between plants still occur. Stalk to stalk variation in terms of shoot apex morphology is known to increase during the last stages of flowering development (Julien, 1971). In this study, at each sampling time point, three different plants (biological replicates) were randomly chosen from each of the SD and LD treatments. As the sampled plants had their apical meristem excised for dissection, the same plant was unable to be sampled again at subsequent time points, thus small differences in developmental growth rate between plants could cause some variation in levels of *ScFT3* expression between biological replicates and between developmental time points; hence the importance of the *P*-values calculated by the REST program which indicate the significance of the differences in relative expression that are observed. The significant induction of *ScFT3* in inducing SD photoperiodic conditions coinciding with observable elongation of the SAM, coupled with its ability to complement the Arabidopsis *ft-10* mutant, provides strong evidence that *ScFT3* is one (of possibly several) *FT* orthologues in sugarcane.

In summary, we describe the current extent of the PEBP gene family in sugarcane which comprises 13 *FT*-like genes, two *MFT-*like genes, and four *TFL*-like genes. These genes all show very high homology to their corresponding genes in *Sorghum* as expected, and also to *FT*-like, *MFT-*like, and *TFL*-like genes in other species such as maize, rice, and Arabidopsis. Analysis of flower-inducing ability in Arabidopsis demonstrated that the sugarcane *ScFT3* gene can function as a floral inducer. Furthermore, a peak of *ScFT3* expression in SD-induced sugarcane plants coincides with the start of the elongation of the SAM (which indicates that induction has occurred). This evidence suggests that *ScFT3* plays a role in floral induction in sugarcane. However, as at least three *Sorghum FT*-like genes have been shown to function as floral promoters in transgenic Arabidopsis, there may be other sugarcane *FT*-like genes that also play a role in floral induction.

## Supplementary data

Supplementary data are available at *JXB* online.

Table S1A and B. Reference numbers for loci and sequences used to construct the gene tree in Fig. 3.

Table S2. Sucest-Fun and Sugarcane Genome Hub contig references.

Table S3. Relative expression of *ScFT3* over the time course calculated using REST.

Fig. S1. Classification of TFL-like genes in sugarcane.

Fig. S2. Sugarcane *ScFT3* sequence used for flowering analysis in transgenic *Arabidopsis*.

Fig. S3. Sugarcane plants grown in the automated photoperiod facility for the developmental time course.

Fig. S4. Representation of the *ScFT12* gene.

Fig. S5. Representation of the *ScFT2* gene.

Fig. S6. Representation of the *ScFT1* gene.

Fig. S7. Representation of the *ScFT4* gene.

Fig. S8. Representation of the *ScFT5* gene.

Fig. S9. Representation of the *ScFT6* gene.

Fig. S10. Representation of the *ScFT7* gene.

Fig. S11. Representation of the *ScFT8* gene.

Fig. S12. Representation of the *ScFT9* gene.

Fig. S13. Representation of the *ScFT10* gene.

Fig. S14. Representation of the *ScFT11* gene.

Fig. S15. Representation of the *ScFT13* gene.

Fig. S16. Representation of the *ScMFT1* gene.

Fig. S17. Representation of the *ScMFT2* gene.

Fig. S18. Representation of the *ScTFL2* gene.

Fig. S19. Representation of the *ScTFL3* gene.

Fig. S20. Representation of the *ScTFL4* gene.

Fig. S21. Expression of *ScFT3* in SDs and LDs over the developmental time course experiment.

erab539_suppl_supplementary_figures_S1-S21_tables_S1-S3

## Data Availability

GenBank accession numbers for all sequences can be found in Supplementary Tables S1A , S1B, and S2, and the reference transcriptome sequences are available at the Dryad Digital Repository https://doi.org/10.5061/dryad.fn2z34tv2; Venail *et al*., 2022. The RNA-seq data have been deposited in the NCBI BioProject database ID PRJNA707146 and can be found at https://www.ncbi.nlm.nih.gov/sra/PRJNA707146. The transcript sequences of the genes *LHY*, *TOC1*, *GI*, and *FKF1* shown in Fig. 1 are deposited in GenBank (submission ID 2435306).
