# Correction to: Analysis of the PEBP gene family and identification of a novel FT orthologue in sugarcane

**DOI:** 10.1093/jxb/erac106

**Published:** 2022-04-26

**Authors:** Julien Venail, Paulo Henrique da Silva Santos, Joao Ricardo Manechini, Leonardo Cardosos Alves, Maximiliano Scarpari, Thais Falcão, Elisson Romanel, Michael Brito, Renato Vicentini, Luciana Pinto, Stephen Derek Jackson

**Affiliations:** School of Life Sciences, University of Warwick, Gibbet Hill, Coventry, UK; Centro de Cana, Instituto Agronômico de Campinas (IAC), Ribeirão Preto, São Paulo, Brazil; Instituto de Biologia, Universidade Estadual de Campinas (UNICAMP), Campinas, São Paulo, Brazil; SUPERBAC Biotechnology Solutions, Londrina, Paraná, Brazil; Centro de Cana, Instituto Agronômico de Campinas (IAC), Ribeirão Preto, São Paulo, Brazil; Departamento de Biotecnologia, Escola de Engenharia de Lorena (EEL), Universidade de São Paulo (USP), São Paulo, Brazil; Departamento de Biotecnologia, Escola de Engenharia de Lorena (EEL), Universidade de São Paulo (USP), São Paulo, Brazil; Instituto de Ciência e Tecnologia, Universidade Federal de São Paulo (UNIFESP), São José dos Campos, São Paulo, Brazil; Instituto de Biologia, Universidade Estadual de Campinas (UNICAMP), Campinas, São Paulo, Brazil; Centro de Cana, Instituto Agronômico de Campinas (IAC), Ribeirão Preto, São Paulo, Brazil; School of Life Sciences, University of Warwick, Gibbet Hill, Coventry, UK

*Journal of Experimental Botany*, erab539, doi:10.1093/jxb/erab539

In the originally published version of this manuscript, there were typographical errors in two authors names. The first name of the 2^nd^ author should read: “Paulo” instead of: “Paolo”. The first name of the 10^th^ author should read: “Luciana” instead of: “Lucia”. These errors have been corrected online.

