# Supplementary material for: Analysis of the PEBP gene family and identification of a novel FLOWERING LOCUS T orthologue in sugarcane
Source: J Exp Bot. 2021 Dec 10;73(7):2035–49. doi: 10.1093/jxb/erab539 (PMC8982381; doi:10.1093/jxb/erab539)
Supplement: erab539_suppl_supplementary_figures_S1-S21_tables_S1-S3 [file erab539_suppl_supplementary_figures_s1-s21_tables_s1-s3.pdf]

Supplementary Figures and Tables.

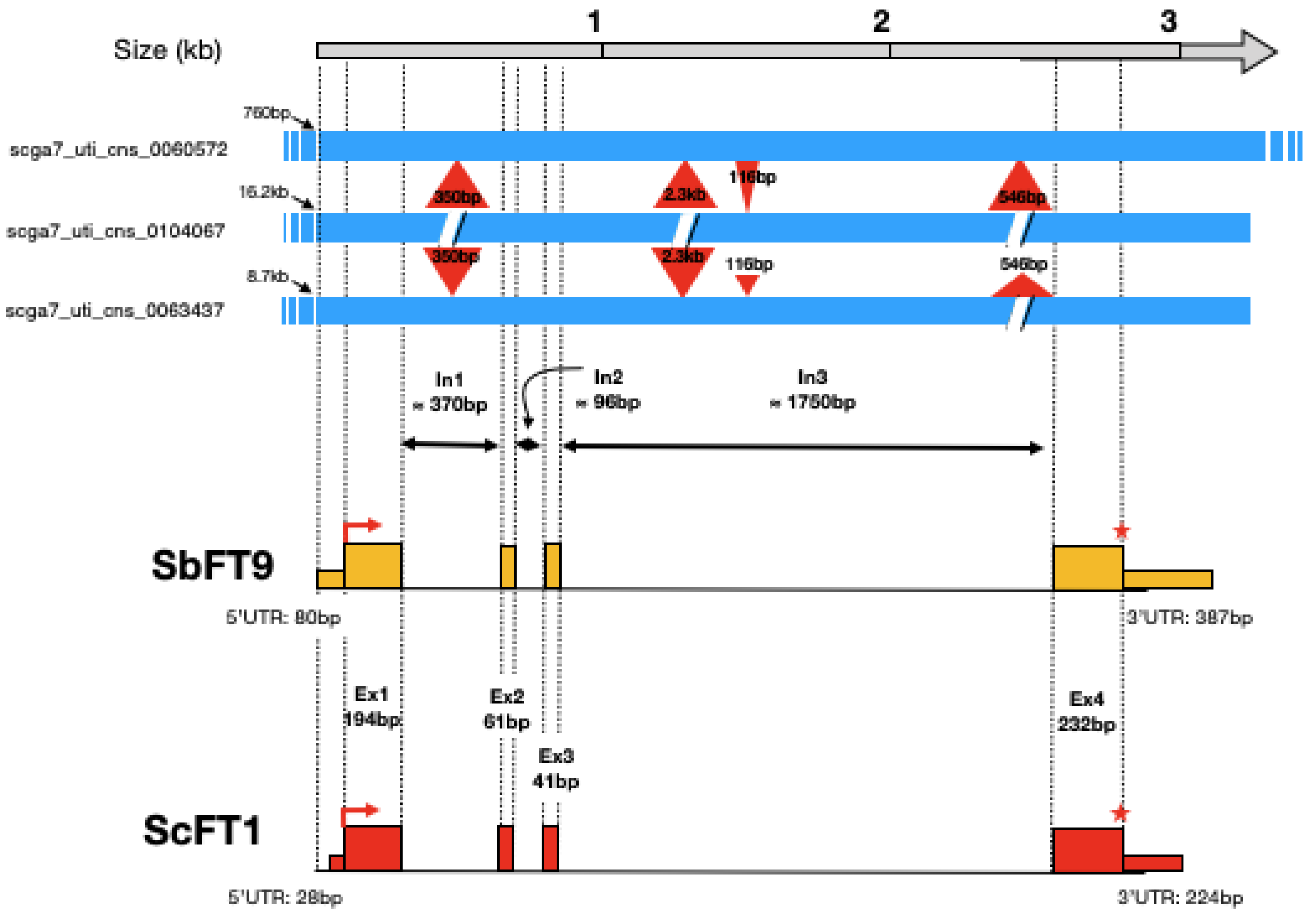

Supplementary Fig. S1

Representation of the ScFT1 gene.
Top half. Alignment of contigs containing the predicted transcript sequence. The top grey line indicates the distances relative to the consensus sequence of the alignment (including insertions). The blue lines indicate matching genomic contig sequences (>90% similarity) with the black arrows indicating the point of alignment of each of the contigs. Indels (>5bp) are represented by red triangles with the point marking deletions in one contig and the opposite side corresponding to insertions in the other. Gaps inserted into the aligned genomic sequences are represented by parallel diagonal lines and indicate large insertions, or regions with low similarity, that have not been included in the graphical representation. Three genomic contigs containing the ScFT1 sequence were identified from the Sucest-Fun database (see Table 1). Alternative versions of the locus are seen in those contigs, differing by several large insertions (up to 2.3kb) in the non-coding sequence. No contigs containing significantly similar ScFT1 sequences were found in the Sugarcane Genome Hub database.

Bottom half. Gene structure of the sugarcane ScFT1 gene.
SbFT9 is the closest homologue in Sorghum, with 95% coding sequence similarity to Sugarcane, a conserved intron-exon structure, 83% and 57% similarity in the 5’UTR and 3’UTR respectively. The red arrows correspond to translational starts, and the red stars to stop codons.

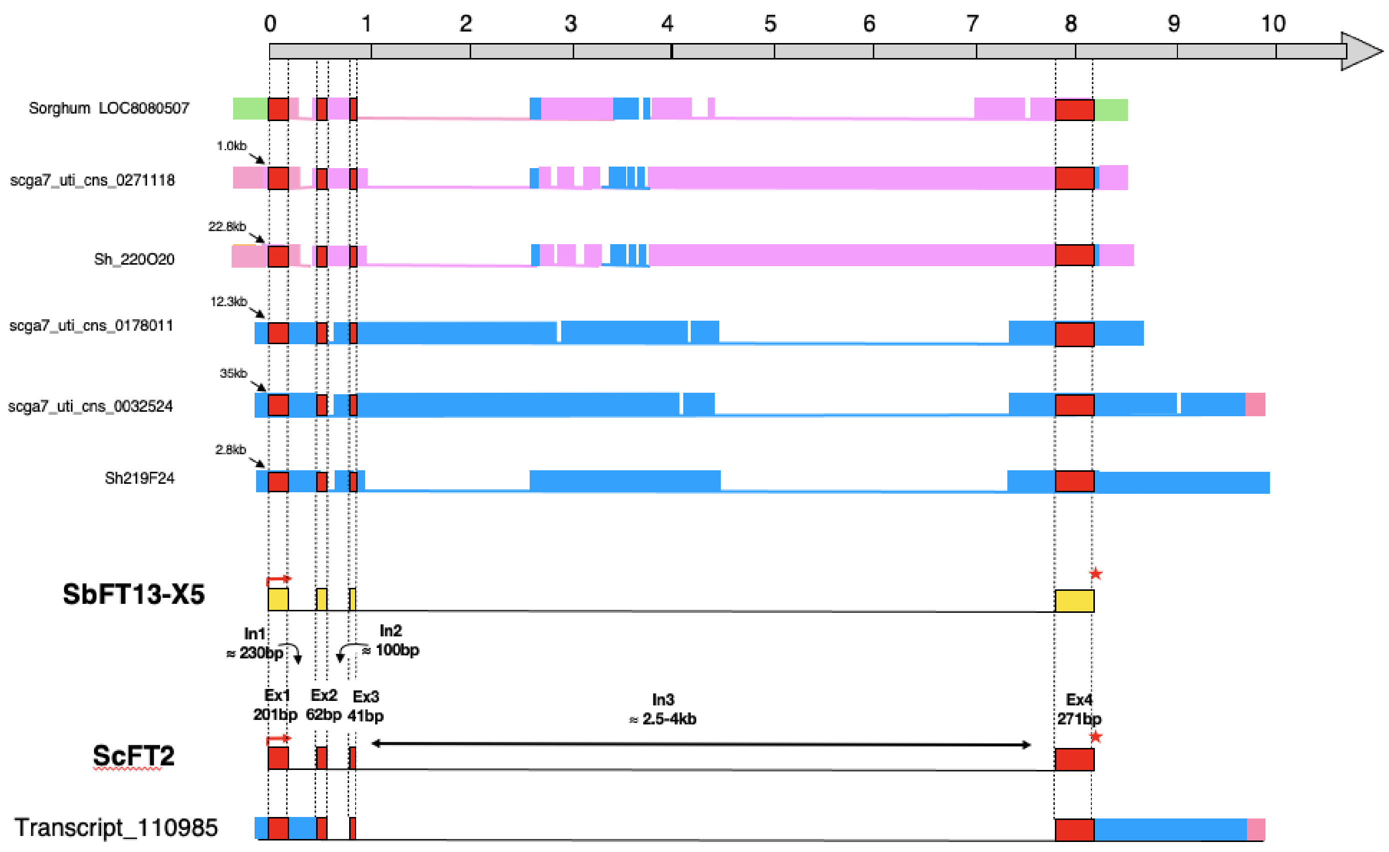

Supplementary Fig. S2

Representation of the ScFT2 gene
Top half. Alignment of contigs containing the predicted transcript sequence. The top grey line indicates the distances relative to the consensus sequence of the alignments with the black arrows indicating the point of alignment of each of the contigs. The blue and pink plain bars indicate matching genomic sequence (>90% similarity) with the thin lines joining those boxes representing gaps in the sequence, other colours indicate divergent sequences. Predicted exons are indicated by red boxes.

In Sorghum, five possible isoforms have been predicted for the SbFT13 locus ("LOC8080507"), however the isoforms X1, X2, X3, X4 have an additional exon located in intron 3, diverging from the canonical 4 exons present in PEBP genes, and the resulting translated proteins have very dissimilar C terminal sequences to any other PEBP gene family members. This not the case for isoform X5, which shows good homology to known PEBP proteins for the full sequence and is therefore the best candidate to use to determine the ORF positions of ScFT2.

Two contigs were found in the Sugarcane Genome Hub database (Sh_219F24 and Sh_220O20) containing sequences homologous to the predicted coding regions in Sorghum SbFT13-X5, however the sugarcane contigs have very different sequences in the non-coding regions with Sh220O20 being similar to the Sorghum gene in these regions, but Sh219F24 being quite different.
The Sucest-Fun database contains another 9 contigs similar to Sh219F24 (only 2 represented here, see Supp table 2 for others), and another 3 similar to Sh220O20. The promoter and 5'UTR of Sh220O20 and related contigs also show some rearrangements when compared to the Sorghum sequence or Sh219F24, possibly due to mobile elements, which could affect the production of a valid transcript.

Bottom half. Gene structure of the sugarcane ScFT2 gene.
The Sorghum SbFT13-X5 isoform is the closest Sorghum homologue to ScFT2, its coding sequence being 90% similar with a conserved intron-exon structure to the sugarcane gene, the sizes of exons (Ex) and introns (In) are indicated. The red arrows correspond to translational starts, the red stars to stop codons.
The sequence of our expressed transcript_110985 matches the sequence of Sh219F24 and several other Sucest-Fun contigs, which are all distinctly recognisable by several SNPs in the 3' region. It is also worth noting that this transcript may not have been fully spliced at the time of detection since it still contains the first intron sequence.

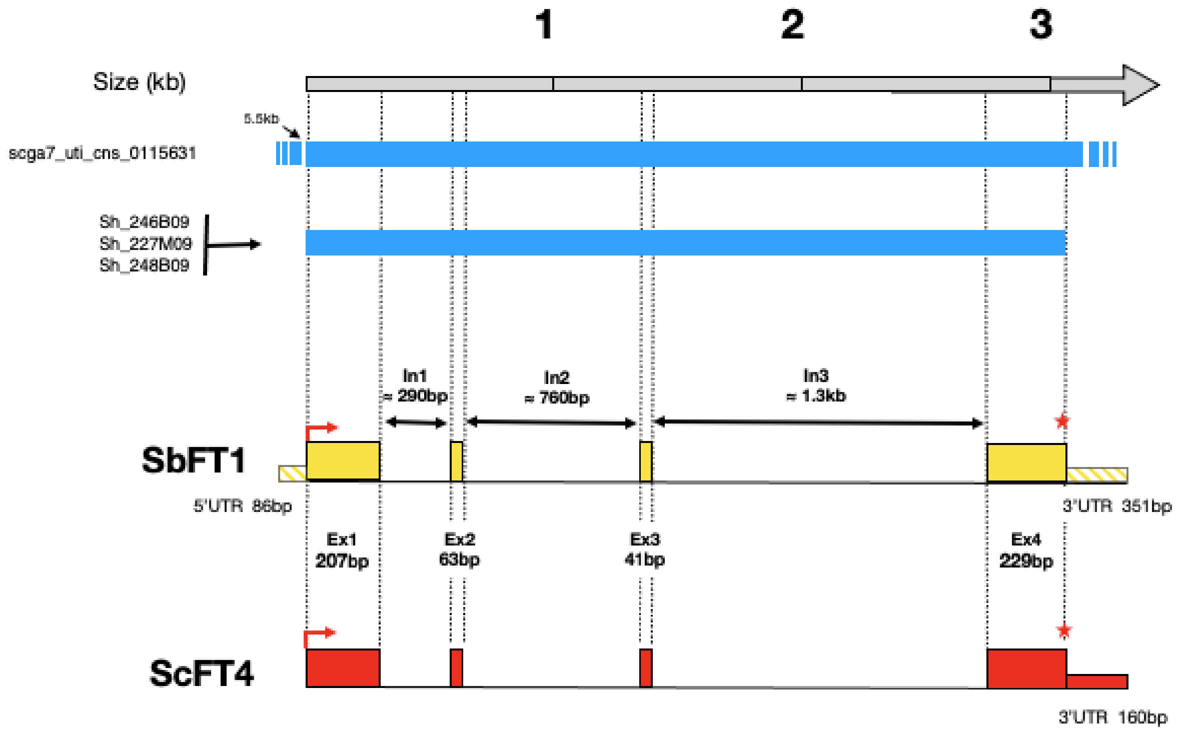

Supplementary Fig. S3

Representation of the ScFT4 gene.
Top half. Alignment of contigs containing the predicted transcript sequence. The top grey line indicates the distances relative to the consensus sequence of the alignment. The blue lines indicate matching genomic sequence (>90% similarity), with the black arrow indicating the start point of alignment of the contig. Contig "scga7_uti_cns_0115631" from the Sucest-Fun database covers the complete ScFT4 transcript. Three contigs containing regions matching to ScFT4 (Sh_246B09, Sh_227M09, and Sh_248B09) are present in the Sugarcane Genome Hub database, their sequences are identical over the coding sequence region.

Bottom half. Gene structure of the sugarcane ScFT4 gene.
SbFT1 is the closest homologue in Sorghum with 98% similarity in its coding sequence but only 20% in the 3'UTR, and it has a conserved intron-exon structure to the sugarcane gene, the sizes of exons (Ex) and introns (In) are indicated. The red arrows correspond to translational starts, and the red stars to stop codons.

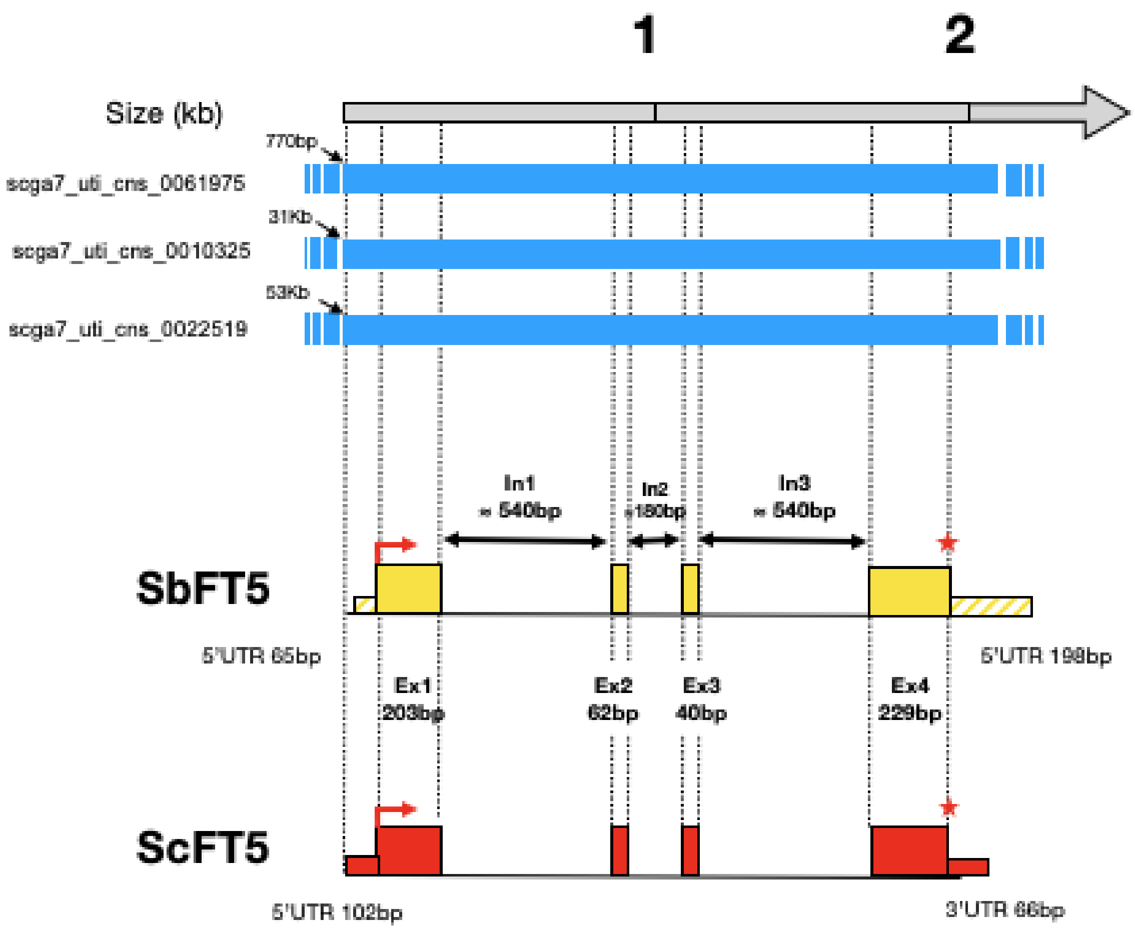

Supplementary Fig. S4

Representation of the ScFT5 gene

Top half. Alignment of contigs containing the predicted transcript sequence. The top grey line indicates the distances relative to the consensus sequence of the alignment. The blue lines indicate matching genomic contig sequences (>90% similarity) with the black arrows indicating the point of alignment of each of the contigs. Three contigs containing the coding sequence from the Sucest-Fun database are shown (see Supp Table 1). No sequence with significant similarity was found in the Sugarcane Genome Hub database.

Bottom half. Gene structure of the sugarcane ScFT5 gene.
SbFT5 is the closest homologue from Sorghum (96.5% coding sequence similarity, but only 35% and 15% in 5’ and 3’UTRs respectively) and it has a conserved intron-exon structure to the sugarcane gene, the sizes of exons (Ex) and introns (In) are indicated. The red arrows correspond to translational starts, and the red stars to stop codons.

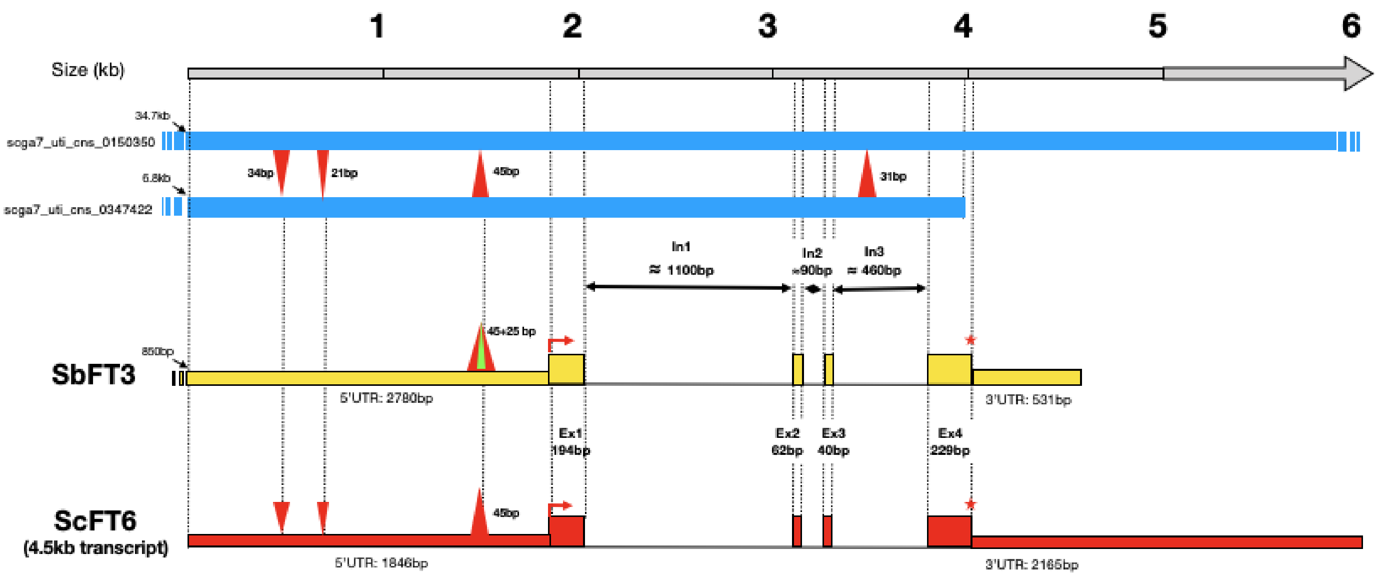

Supplementary Fig. S5

Representation of the ScFT6 gene

Top half. Alignment of contigs containing the predicted transcript sequence. The top grey line indicates the distances relative to the consensus sequence of the alignment (including insertions). The blue lines indicate matching genomic sequence (>90% similarity) with the black arrows indicating the point of alignment of each of the contigs. Indels (>5bp) are represented by red rectangles with the pointed end marking a deletion in one contig and the opposite side corresponding to an insertion in the other.
The sequence of the ScFT6 RNAseq transcript detected in this study is present in contigs “scga7_uti_cns_0150350” and “scga7_uti_cns_0347422”, but with the latter missing the 3’end of the gene. No sequence with sufficient similarity was found in the Sugarcane Genome Hub database.

Bottom half. Gene structure of the sugarcane ScFT6 gene.
SbFT3 is the closest homologue in Sorghum with 97% coding sequence similarity (85% and 88% in the 5’ and 3’UTR regions respectively) and a conserved intron-exon structure to the sugarcane gene, the sizes of exons (Ex) and introns (In) are indicated. The red arrows correspond to translational starts, and the red stars to stop codons.
The long 5’ and 3’ UTRs include a number of noticeable indels: 300bp upstream of the start codon, a 45bp insertion is present in contig “scga7_uti_cns_0347422” and the ScFT6 transcript, which is also present in the Sorghum SbFT3 at the same position. In SbFT3 however another 25bp insertion is symmetrically nested within the 45bp insertion, possibly the result of mobile element activity at this site following the split between the sorghum and sugarcane lineages.
A recently published transcript (Genbank ref MN458470.1 from cultivar SP80-3280) of ScFT6 is mostly identical to our ScFT6 transcript, The only difference in the coding sequence being a single base pair change (T to G at position 416 of MN458470.1) which causes an amino acid change from V to F at position 129 (a variable amino acid position across all FT-like genes).

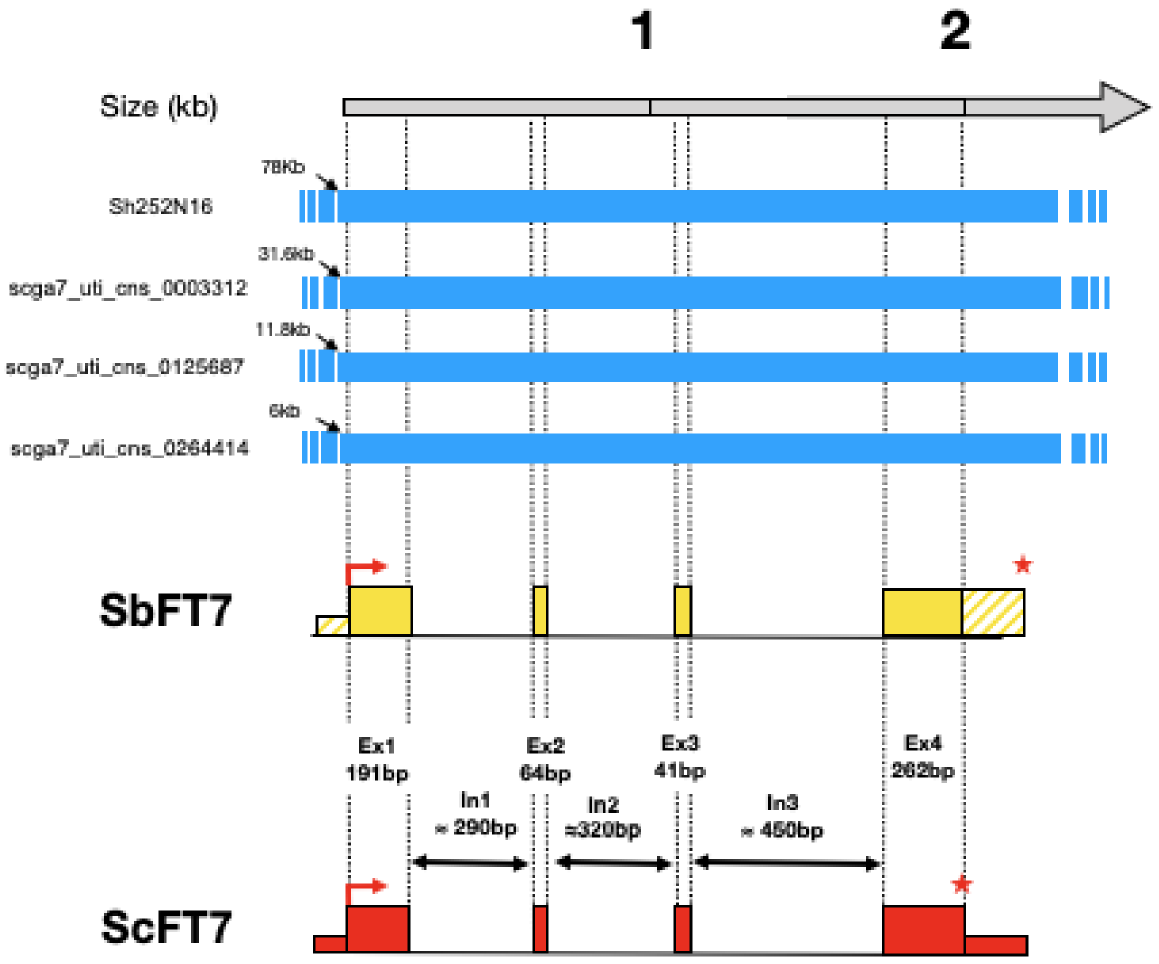

Supplementary Fig. S6

Representation of the ScFT7 gene
Top half. Alignment of contigs containing the predicted transcript sequence. The top grey line indicates the distances relative to the consensus sequence of the alignment. The blue lines indicate matching genomic contig sequences (>90% similarity) with the black arrows indicating the point of alignment of each of the contigs. The sequence of ScFT7 was obtained by searching for a homolog of SbFT7 in the Sugarcane Genome Hub database. Subsequently, three contigs containing the gene were then found in the Sucest-Fun database. Their coding sequence has 76.8% similarity with most of the difference accounted for by sequence divergence in the last exon, introducing a premature stop codon 144bp before the 3' end of SbFT7.

Bottom half. Gene structure of the sugarcane ScFT7 gene.
ScFT7 has a 97.8% protein sequence similarity to SbFT7, with most the difference explained by the shorter C-terminus of ScFT7 (48aa less). Apart from the last exon the two genes have a conserved intron-exon structure the sizes of exons (Ex) and introns (In) are indicated. The 5' & 3' UTRs are depicted but show low sequence similarity to sorghum (<50%) and have not being confirmed by sequencing of a transcript. The red arrows correspond to translational starts, and the red stars to stop codons.

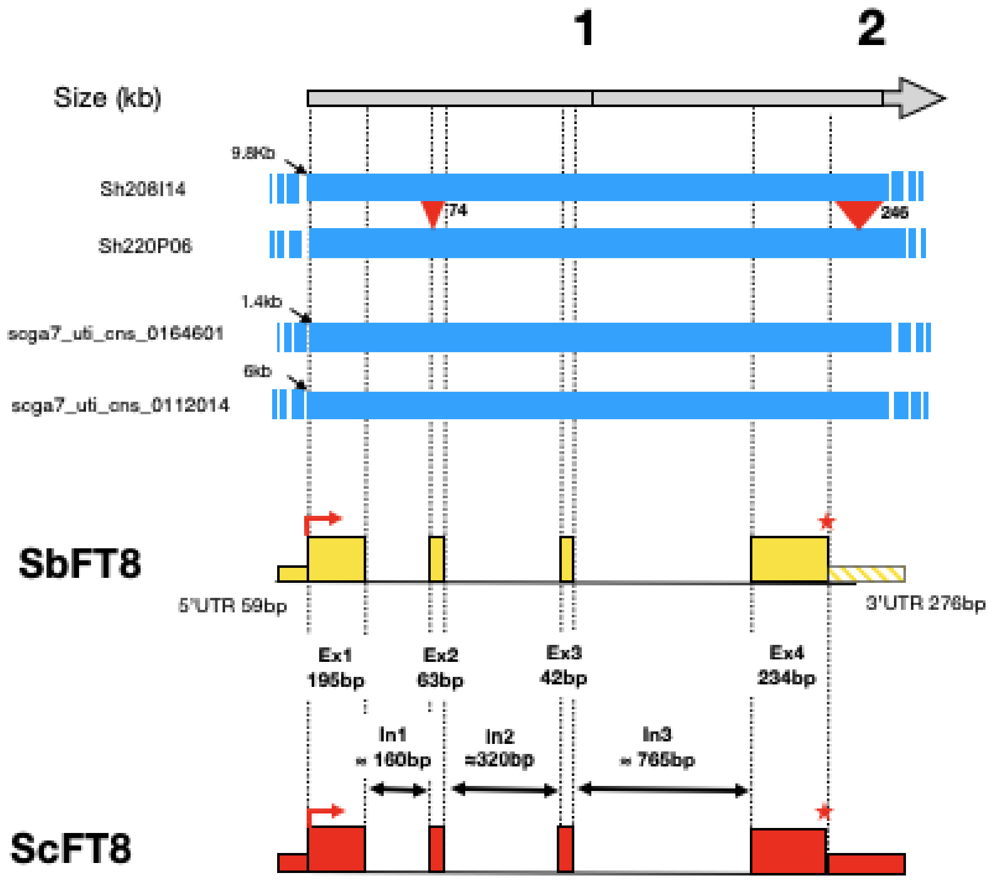

Supplementary Fig. S7

Representation of the ScFT8 gene

Top half. Alignment of contigs containing the predicted transcript sequence. The top grey line indicates the distances relative to the consensus sequence of the alignment (including insertions). The blue lines indicate matching genomic contig sequences (>90% similarity) with the black arrows indicating the point of alignment of each of the contigs. The sequence of ScFT8 was obtained by searching for a homolog of SbFT8 in the Sugarcane Genome Hub database. The BAC “Sh208I14" contains the full gene, whereas “Sh220P06” contains a deletion of 74bp removing the second exon as well as a second deletion of 246bp in the 3’UTR. Two other contigs containing the gene were found in the Sucest-Fun database, both matching the complete “Sh208I14” sequence.

Bottom half. Gene structure of the sugarcane ScFT8 gene.
ScFT8 has a 96% coding sequence similarity to SbFT8 and a conserved intron-exon structure, the sizes of exons (Ex) and introns (In) are indicated. The red arrows correspond to translational starts, and the red stars to stop codons. The 5’ and 3’ UTRs are depicted but have not being confirmed by sequencing of a transcript. The 3’UTR sequence from sugarcane shows low similarity to sorghum.

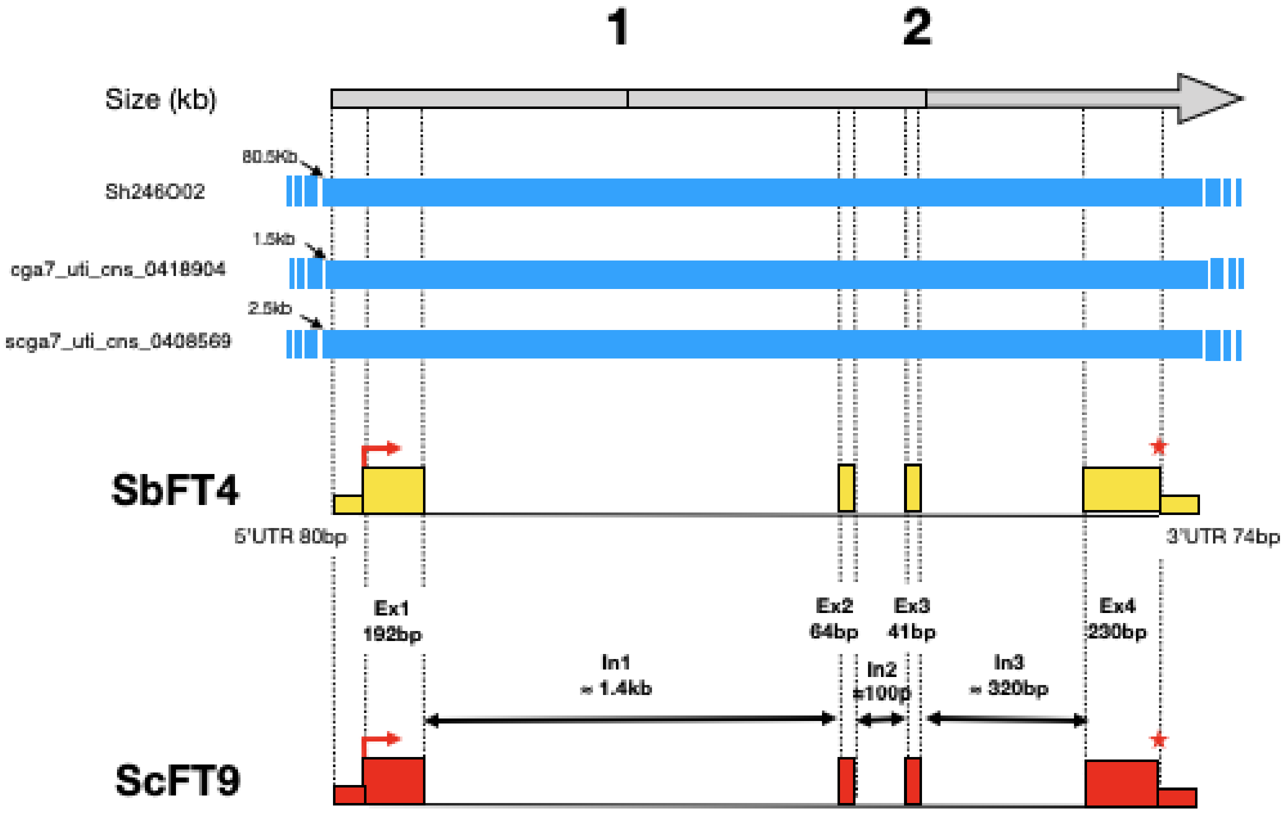

Supplementary Fig. S8

Representation of the ScFT9 gene
Top half. Alignment of contigs containing the predicted transcript sequence. The top grey line indicates the distances relative to the consensus sequence of the alignment. The blue lines indicate matching genomic contig sequences (>90% similarity) with the black arrows indicating the point of alignment of each of the contigs. The sequence of ScFT9 was obtained by searching for a homologue of SbFT4 in the Sugarcane Genome Hub database. Subsequently two contigs containing the gene (cga7_uti_cns_0418904 and scga7_uti_cns_0408569) were also found in the Sucest-Fun database.

Bottom half. Gene structure of the sugarcane ScFT9 gene.
ScFT9 has a 95% sequence similarity to SbFT4 over the full gene sequence (coding and non-coding), and a conserved intron-exon structure, the sizes of exons (Ex) and introns (In) are indicated. The red arrows correspond to translational starts, and the red stars to stop codons. The 5' and 3' UTRs are depicted but have not being confirmed by sequencing of a transcript.

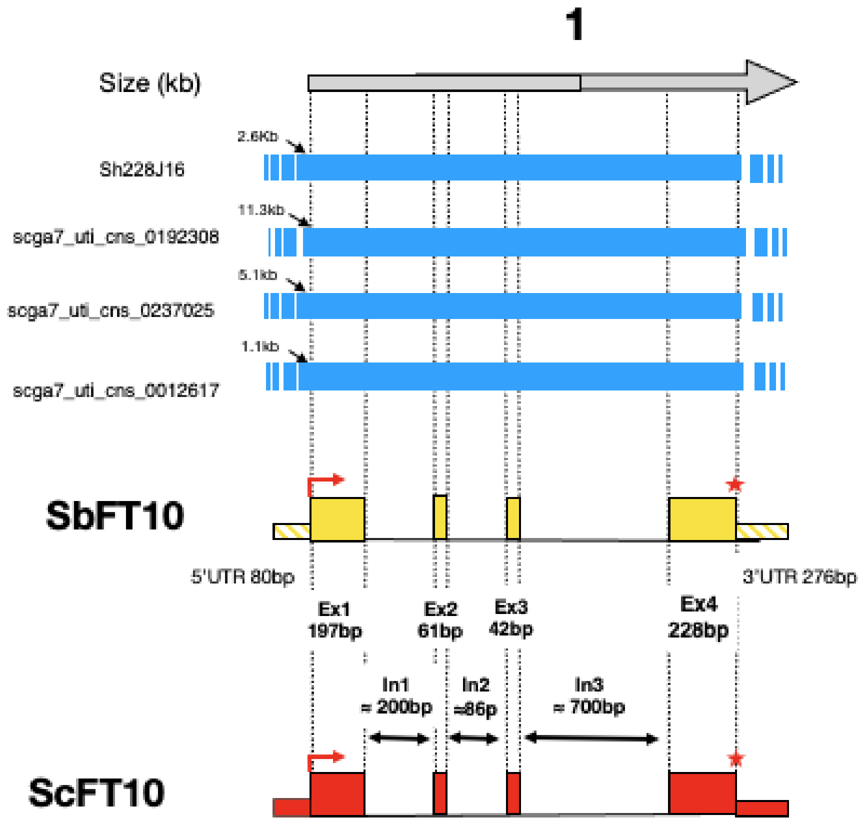

Supplementary Fig. S9

Representation of the ScFT10 gene
Top half. Alignment of contigs containing the predicted transcript sequence. The top grey line indicates the distances relative to the consensus sequence of the alignment. The blue lines indicate matching genomic contig sequences (>90% similarity) with the black arrows indicating the point of alignment of each of the contigs. The sequence of ScFT10 was obtained by searching for a homolog of SbFT10 in the Sugarcane Genome Hub database. Subsequently three contigs containing the gene were found in the Sucest-Fun database.

Bottom half. Gene structure of the sugarcane ScFT10 gene.
ScFT10 has a 93.7% protein sequence (77.7% coding sequence) similarity to SbFT10, and a conserved intron-exon structure, the sizes of exons (Ex) and introns (In) are indicated. The red arrows correspond to translational starts, and the red stars to stop codons. The 5’ and 3’ UTRs are depicted, but show low sequence similarity to sorghum (<50%) and have not being confirmed by sequencing of a transcript.

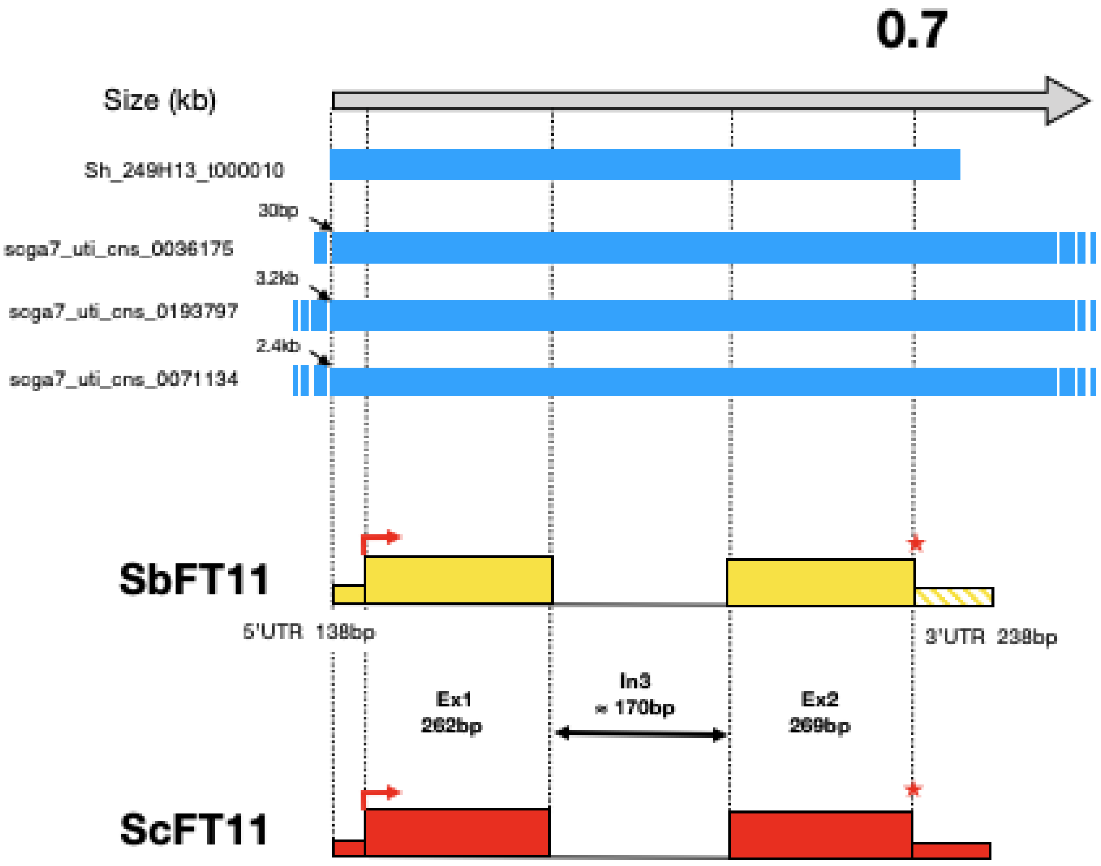

Supplementary Fig. S10

Representation of the ScFT11 gene
Top half. Alignment of contigs containing the predicted transcript sequence. The top grey line indicates the distances relative to the consensus sequence of the alignment. The blue lines indicate matching genomic contig sequences (>90% similarity) with the black arrows indicating the point of alignment of each of the contigs. The sequence of ScFT11 was obtained by searching for a homolog of SbFT11, "Sh_249H13_t000010" was identified in the Sugarcane Genome Hub database and then three other contigs that include the full gene were found in the Sucest-Fun database.

Bottom half. Gene structure of the sugarcane ScFT11 gene.
ScFT9 has a 94% coding sequence similarity to SbFT11 and a conserved intron-exon structure, the sizes of exons (Ex) and introns (In) are indicated. The red arrows correspond to translational starts, the red stars to stop codons. The 3' and 5' UTRs are depicted, and show high sequence similarity to sorghum, but have not being confirmed by sequencing of a transcript.

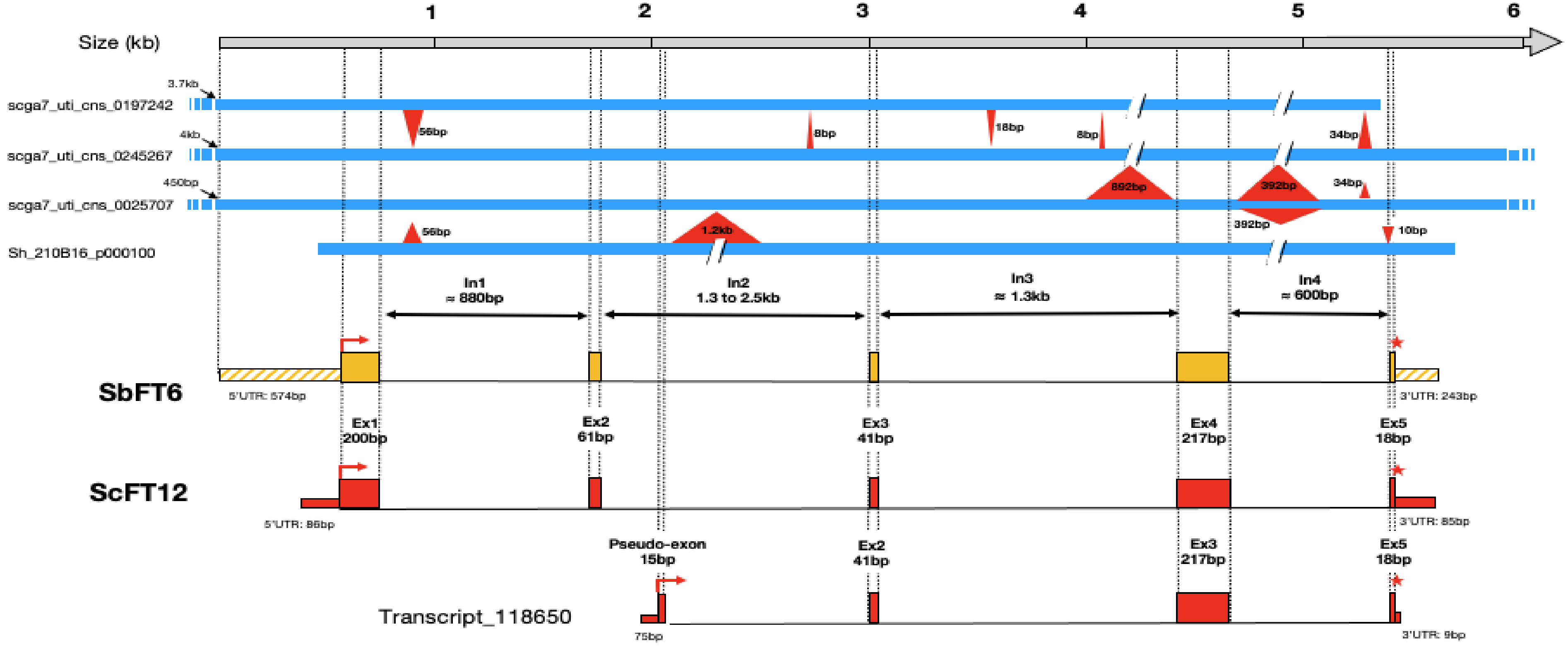

Supplementary Fig. S11

Representation of the ScFT12 gene

Top half. Alignment of contigs containing the predicted transcript sequence. The top grey line indicates the distances relative to the consensus sequence of the alignment (including insertions). The blue lines indicate matching genomic contig sequences (>90% similarity) with the black arrows indicating the point of alignment of each of the contigs. Indels (>5bp) are represented by red rectangles with the pointed end marking a deletion in one contig and the opposite side corresponding to an insertion in the other. Gaps inserted into the aligned genomic sequences are represented by parallel diagonal lines and indicate large insertions, or regions lacking sequence similarity, that have not been included in the graphical representation.
The ScFT12 sequence is present in 3 contigs from the Sucest-Fun database (“scga7_uti_cns_0197242”, “scga7_uti_cns_0245267”, and “scga7_uti_cns_0025707”), and one sequence in the Sugarcane Genome Hub database (“Sh_210B16_p000100”) which is likely to represent the full length ScFT12 coding sequence. A large indel and some single base pair variations are present in the 4th intron of the ScFT12 gene. The rearrangements in this region could explain the presence of a 4th intron, which is unusual within the PEBP domain family members.

Bottom half. Gene structure of the sugarcane ScFT12 gene.
SbFT6 is the closest homologue in Sorghum with 95% coding sequence similarity to sugarcane and a conserved intron-exon structure, the sizes of exons (Ex) and introns (In) are indicated. The red arrows correspond to translational starts, the red stars to stop codons. The 5’ and 3’ UTRs of ScFT12 are depicted but show low similarity (<40%) to SbFT6.
Transcript 118650 identified via RNAseq in this study is likely to be a partial or mis-spliced mRNA. It contains an in-frame start codon that could lead to a transcript with an alternative first exon. The SbFT6 locus (LOC8054807) is predicted to contain several splice variants for this gene including a matching predicted splice variant to the sugarcane Transcript_118650 from this study. These Sorghum variants (XM021454650.1, XM021454649.1 and XM021454648.1) have the same pseudo-exon with a valid ORF, around 1.5kb downstream of the ATG, that leads to a truncated transcript corresponding to only the 3’ part of the gene after the 2nd exon, which misses around 60 amino acids of the N-terminal of the protein. Similar variation has been reported for the maize homologue (Zcn18; Danilevskaya et al. 2008).

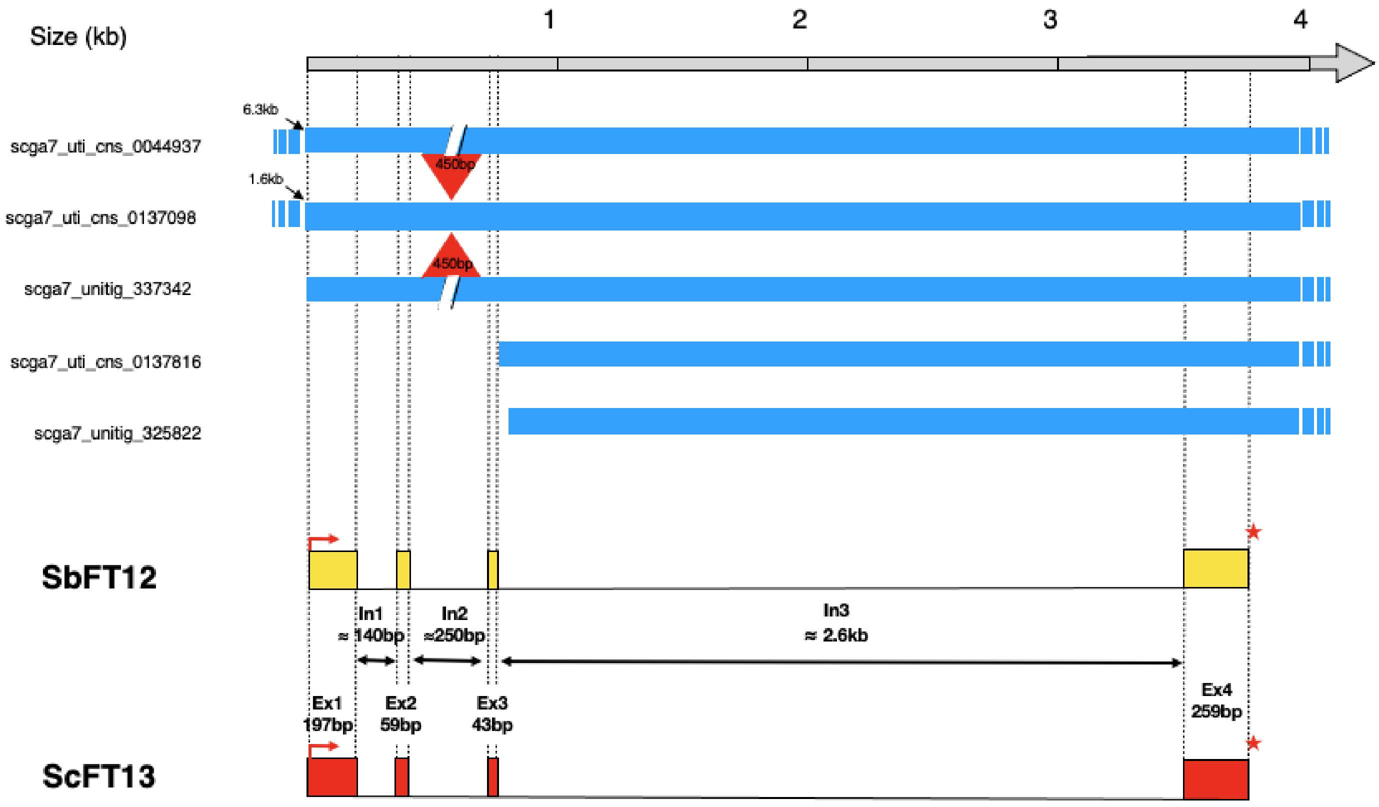

Supplementary Fig. S12

Representation of the ScFT13 gene
Top half. Alignment of contigs containing the predicted transcript sequence. The top grey line indicates the distances relative to the consensus sequence of the alignment (including insertions). The blue lines indicate matching genomic contig sequences (>90% similarity) with the black arrows indicating the point of alignment of each of the contigs. Gaps inserted into the aligned genomic sequences are represented by parallel diagonal lines and indicate large insertions. The sequence of ScFT13 was obtained by searching for a homolog of SbFT12, five contigs containing all, or parts, of the gene were found in the Sucest-Fun database. No homologous sequence was found in the Sugarcane Genome Hub database.

Bottom half. Gene structure of the sugarcane ScFT13 gene.
SbFT12 and ScFT13 share 97% protein sequence similarity and a conserved intron-exon structure, the sizes of exons (Ex) and introns (In) are indicated. The red arrows correspond to translational starts, the red stars to stop codons.

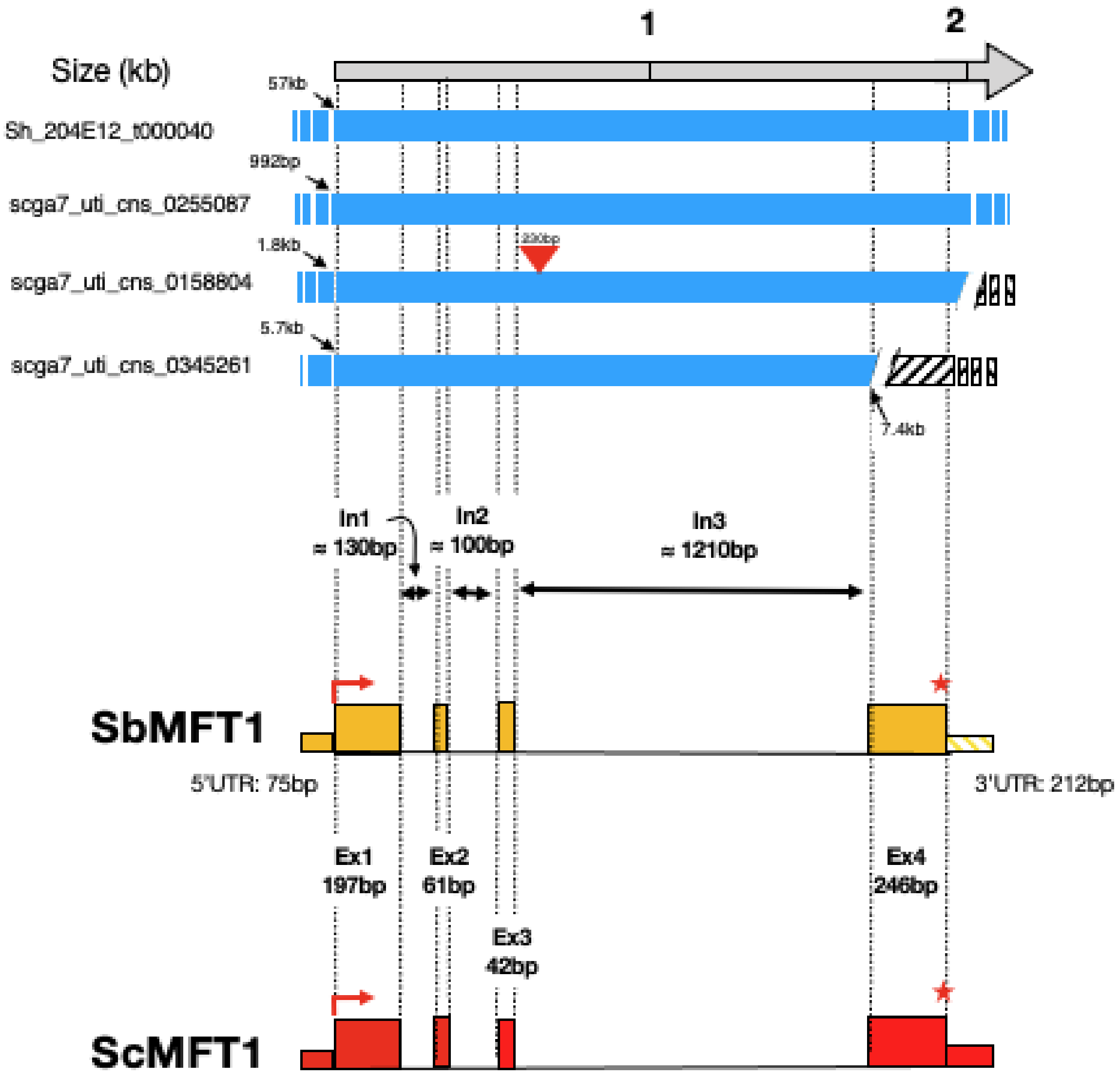

Supplementary Fig. S13

Representation of the ScMFT1 gene
Top half. Alignment of contigs containing the predicted transcript sequence.
The top grey line indicates the distances relative to the consensus sequence of the alignment (including insertions). The blue lines indicate matching genomic sequence (>90% similarity) with the black arrows indicating the point of alignment of each of the contigs. Diagonal cross lines followed by hatched line indicate the end of homology amongst contigs.
The Sugarcane Genome Hub database contains the full-length predicted transcript (Sh_204E12_t000040).
Three contigs from the Sucest-Fun database containing this coding sequence are represented (see Supp Table 2 for contig details).
Scga7_uti_cns_0255087 contains the full gene, and its sequence similarity to Sh_204E12_t000040 extends for several kilobases in the 5' and 3' direction.
Scga7_uti_cns_0158804 sequence diverges100bp after the stop codon.
Scga7_uti_cns_0345261 diverges before the end of the gene, 1.7kb after the start codon.

Bottom half. Gene structure of the sugarcane ScMFT1 gene. SbMFT1 is the closest homologue in Sorghum, its coding sequence being 95% similar (but only 5% similarity in the 3'UTR) and it has a conserved intron-exon structure to the sugarcane gene, the sizes of exons (Ex) and introns (In) are indicated. The red arrows correspond to translational starts, and the red stars to stop codons. The 3' and 5' UTRs are depicted but have not being confirmed by sequencing of a transcript.

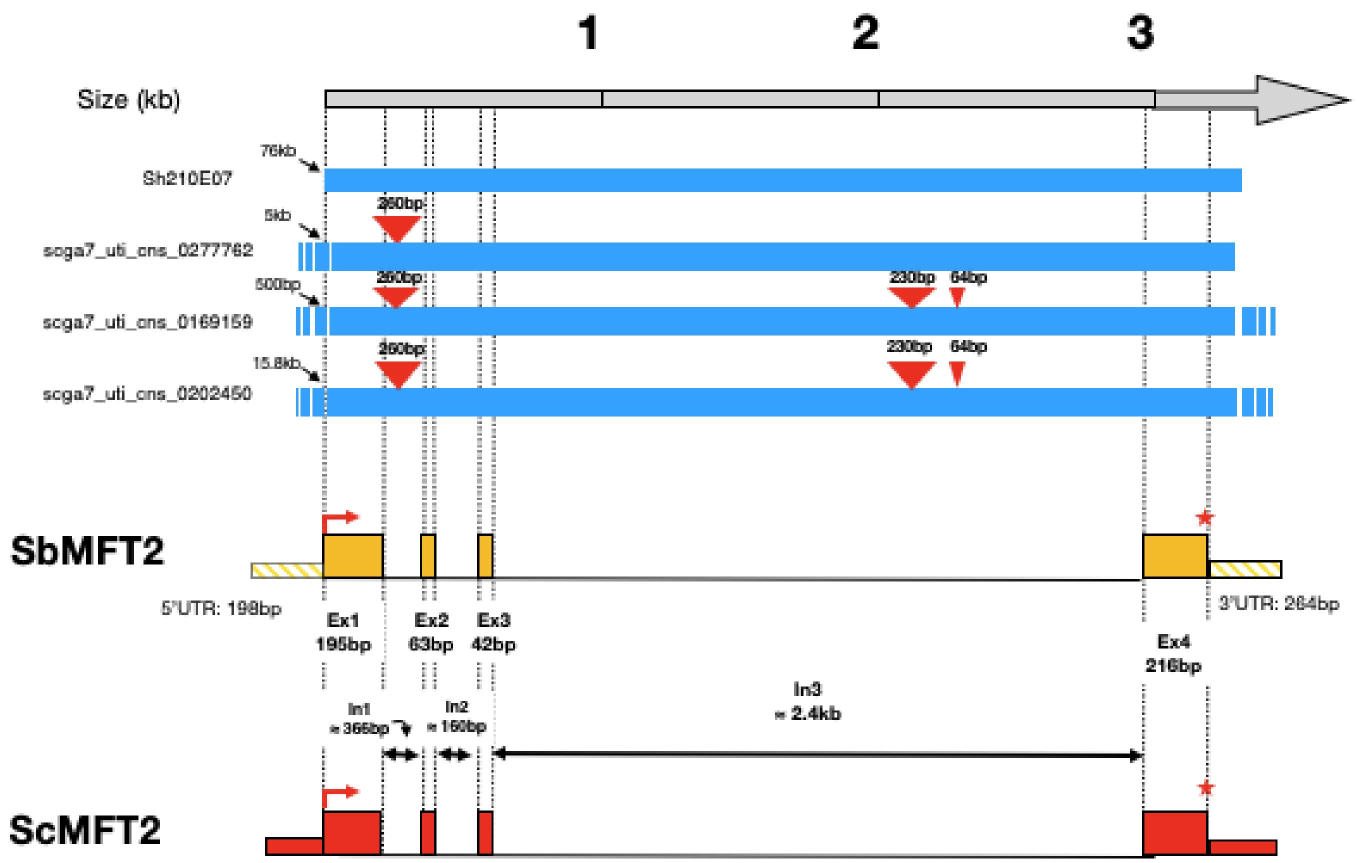

Supplementary Fig. S14

Representation of the ScMFT2 gene
Top half. Alignment of contigs containing the predicted transcript sequence.
The top grey line indicates the distances relative to the consensus sequence of the alignment (including insertions). The blue lines indicate matching genomic sequence (>90% similarity) with the black arrows indicating the point of alignment of each of the contigs.
The Sugarcane Genome Hub database contains the full-length predicted transcript (Sh_210E07), three other contigs containing the gene were also found in the Sucest-Fun database, all of them containing the same deletion of 260bp in the first intron, and two of them further deletions in the third intron, but with no change in their coding sequence.

Bottom half. Gene structure of the sugarcane ScMFT2 gene. ScMFT2 has 99.5% protein sequence similarity to SbMFT2, and 97% coding sequence similarity and a conserved intron-exon structure. The sizes of exons (Ex) and introns (In) are indicated. Their full sequences (including UTRs) are however very divergent (only 40% similarity). The red arrows correspond to translational starts, and the red stars to stop codons.

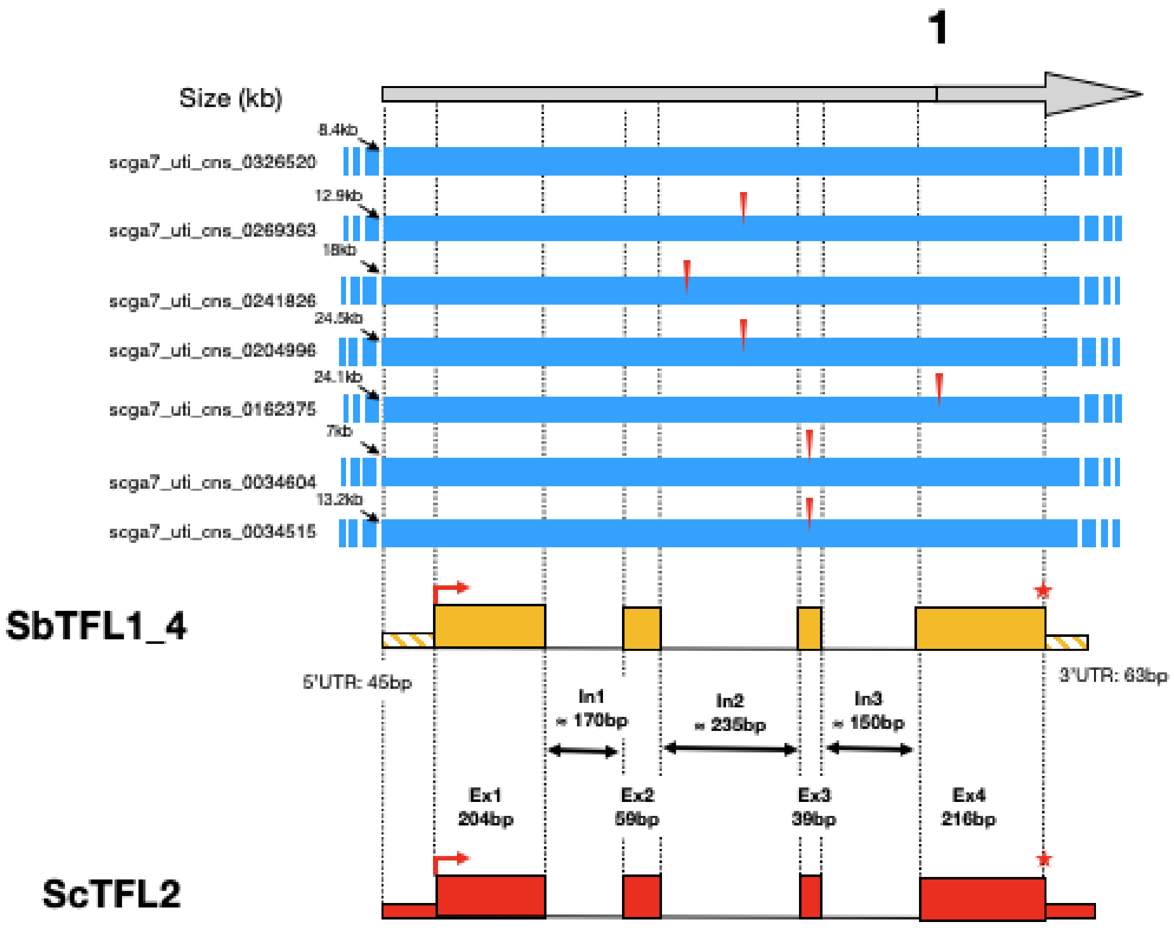

Supplementary Fig. S15

Representation of the ScTFL2 gene
Top half. Alignment of contigs containing the predicted transcript sequence.
The top grey line indicates the distances relative to the consensus sequence of the alignments (including insertions). The blue lines indicate matching genomic sequence (>90% similarity) with the black arrows indicating the point of alignment of each of the contigs and the small red triangles indicating single nucleotide changes.
The 7 contigs containing ScTFL2 were found by screening the Sucest-Fun database using the sequence of SbTFL1_4. No matching gene was found in the Sugarcane genome hub.
The first four contigs shown here have an identical coding region sequences, the changes seen in the other two could be sequencing errors.

Bottom half. Gene structure of the sugarcane ScTFL2 gene. ScTFL2 has 97.8% coding sequence similarity to SbTFL1_4, with a conserved intron-exon structure. The sizes of exons (Ex) and introns (In) are indicated. The red arrows correspond to translational starts, the red stars to stop codons. The 3’ and 5’ UTRs of ScTFL2 are depicted but show low similarity to Sorghum and haven’t confirmed by sequencing of a transcript.

Supplementary Fig. S16

Representation of the ScTFL3 gene

Top half. Alignment of contigs containing the predicted transcript sequence.
The top grey line indicates the distances relative to the consensus sequence of the alignments (including insertions). The blue lines indicate matching genomic sequence (>90% similarity) with the black arrows indicating the point of alignment of each of the contigs.
5 contigs containing the ScTFL3 gene were found in the Sucest-Fun database (3 shown here).

Bottom half. Gene structure of the sugarcane ScTFL3 gene. It's closest homologue in Sorghum is "SbTFL1_1" which has 98% coding sequence similarity, with a conserved intron-exon structure. The sizes of exons (Ex) and introns (In) are indicated. The 3' and 5' UTRs are depicted, and show high sequence similarity to Sorghum, but have not being confirmed by sequencing of a transcript. The red arrows correspond to translational starts, the red stars to stop codons.

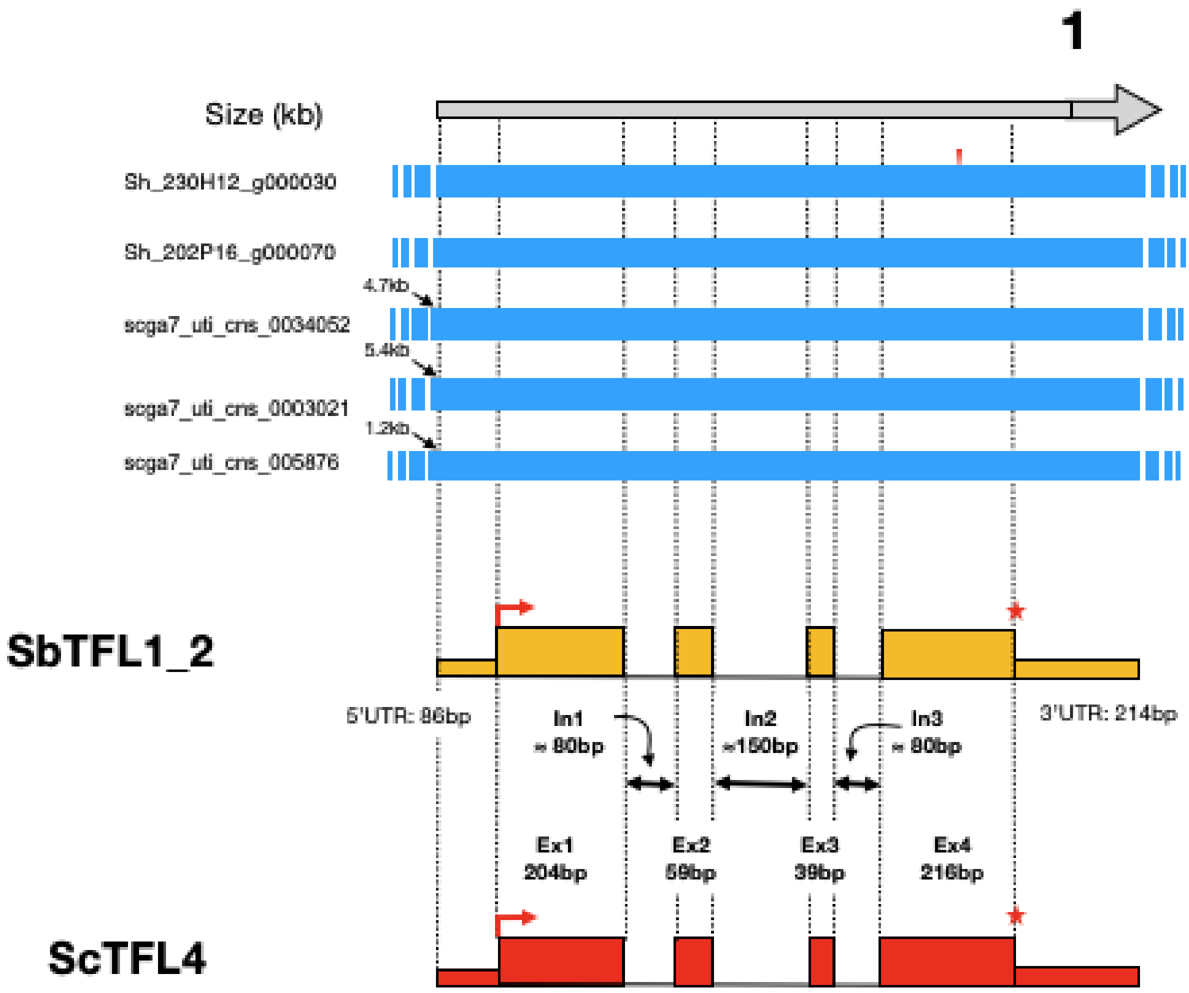

Supplementary Fig. S17

Representation of the ScTFL4 gene

Top half. Alignment of contigs containing the predicted transcript sequence.

The top grey line indicates the distances relative to the consensus sequence of the alignments (including insertions). The blue lines indicate matching genomic sequence (>90% similarity) with the black arrows indicating the point of alignment of each of the contigs.

Two near identical predicted transcripts related to TFL-like genes were found in the Sugarcane Genome Hub database (“Sh_230H12_t000030” and “Sh_202P16_t000070”; 99.5% protein sequence similarity with only a single base pair difference indicated by small red triangle). Three matching contigs were found in the Sucest-Fun database containing the same sequence.

Bottom half. Gene structure of the sugarcane ScTFL4 gene.

It’s closest homologue in Sorghum is “SbTFL1_2” which has 76% coding sequence similarity (95.4% for the protein sequence, 92% for the 5’UTR and 3’UTR), with a conserved intron-exon structure. The sizes of exons (Ex) and introns (In) are indicated. ScTFL4 has a 95.5% protein sequence similarity to ScTFL1, with 11aa differences where their sequences overlap, and ScTFL1 having a 18aa shorter N terminal. The red arrows correspond to translational starts, the red stars to stop codons.

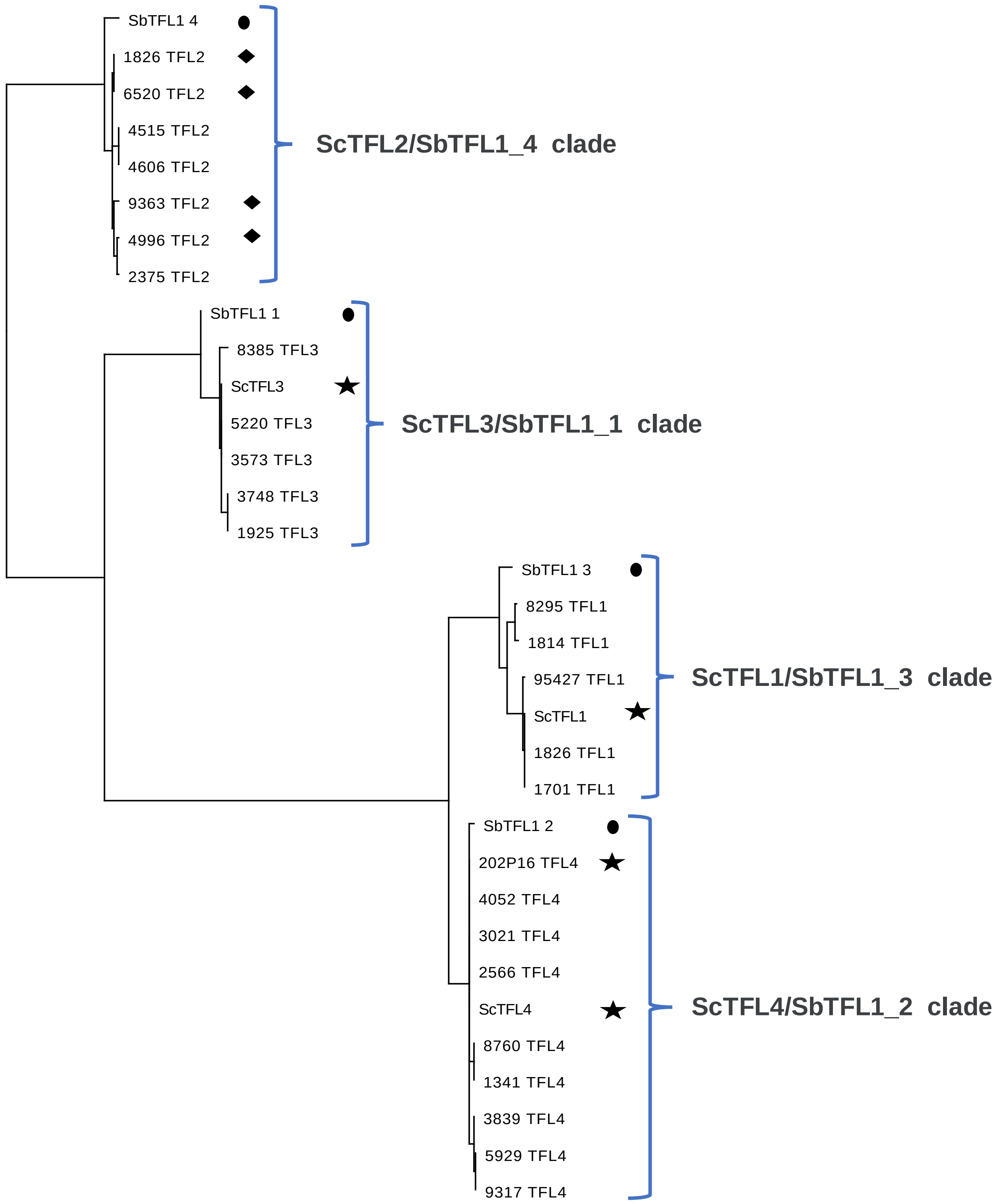

Supplementary Fig. S18

Classification of TFL-like genes in sugarcane
This gene tree was obtained by aligning nucleotide coding sequences for TFL-like genes found in publicly available databases. The 4 TFL-like genes from *Sorghum* (SbTFL1_1 to SbTFL1_4) are indicated with dots. The published sequences from sugarcane (ScTFL1, ScTFL3 & ScTFL4) are indicated with stars, in the case of ScTFL2 the four entries indicated by diamonds have identical sequence, and are the most parsimonious to SbTFL1-4. They were therefore chosen as the reference sequence. The three other entries have only a single nucleotide difference each.
The various copies found in the Sucest-Fun database are designated by the 4 last digits of their contig number (see Supp Table 2 for full reference). ScTFL_202_P16 is the only TFL-like gene found in the Sugarcane Genome Hub database.
All sequences obtained from contigs fall within defined single clades containing one of the sugarcane TFL-like genes (ScTFL1 to ScTFL4), suggesting they are redundant copies of the same gene with little variation. They were therefore considered as a single gene in this study. Single nucleotide variation observed between entries within individual clade of each gene have very little effect on the amino acid sequence.

A.

```
Synthetic ScFT3 sequence  ATGCAGAGGGGGACCCTCTTGTGGTGGGACGTATCATCGGAGATGTAGTAGATCCGTTCGTGCGAAGA
Sugarcane ScFT3sequence- ATGCAGCGCGGGGACCCGCTGGTGGTGGGGCGCATCATCGGCGACGTGGTGGACCCCTTCGTGCGCCGG

GTACCGTTGCGTGTCGCTTATGCTGCCCGTGAAATCAGCAACGGTTGCGAACTCCGTCCTTCAGCAATCGCAGATCAGCCACGCGTCGAGGTCGGCGGAC
GTGCCGCTCCGCGTCGCCTACGCCGCGCGCGAGATCTCCAACGGCTGCGAGCTCAGGCCCTCCGCCATCGCCGACCAGCCGCGCGTCGAGGTCGGCGGAC

CCGACATGCGCACCTTCTACACCCTCGTGATGGTGGATCCTGATGCGCCAAGCCCCAGCGATCCCAACCTCAGGGAGTACCTGCACTGGCTGGTCACTGA
CCGACATGCGCACCTTCTACACCCTCGTGATGGTGGATCCTGATGCGCCAAGCCCCAGCGATCCCAACCTCAGGGAGTACCTGCACTGGCTGGTCACTGA

CATTCCGGCGACGACTGGAGTTTCTTTTGGGACTGAGGTTGTGTGCTACGAGAGCCCACGGCCGGTGCTGGGAATCCACAGGATAGTGTTTCTGCTCTTC
CATTCCGGCGACGACTGGAGTTTCTTTTGGGACTGAGGTTGTGTGCTACGAGAGCCCACGGCCGGTGCTGGGAATCCACAGGATAGTGTTTCTGCTCTTC

CAACAGCTCGGCCGGCAGACGGTCTACGCCCCAGGGTGGCGGCAGAACTTCAGCACCCGTGACTTCGCCGAGCTCTACAACCTCGGCTTGCCGGTCGCCG
CAACAGCTCGGCCGGCAGACGGTCTACGCCCCAGGGTGGCGGCAGAACTTCAGCACCCGTGACTTCGCCGAGCTCTACAACCTCGGCTTGCCGGTCGCCG

CTGTCTACTTCAACTGCCAAAGGGAGTCCGGAACTGGTGGGAGAAGAATGTGA
CTGTCTACTTCAACTGCCAAAGGGAGTCCGGAACTGGTGGGAGAAGAATGTGA
```

B.

```
1      MQRGDPLVVGRIIGDVVDPFVRRVPLRVAYAAREISNGCELRPSAIADQPRVEVGGPDMR   60
       ||||||||||||||||||||||||||||||||||||||||||||||||||||||||||||
1      MQRGDPLVVGRIIGDVVDPFVRRVPLRVAYAAREISNGCELRPSAIADQPRVEVGGPDMR   60

61     TFYTLVMVDPDAPSPSDPNLREYLHWLVTDIPATTGVSFGTEVVCYESPRPVLGIHRIVF   120
       ||||||||||||||||||||||||||||||||||||||||||||||||||||||||||||
61     TFYTLVMVDPDAPSPSDPNLREYLHWLVTDIPATTGVSFGTEVVCYESPRPVLGIHRIVF   120

121    LLFQQLGRQTVYAPGWRQNFSTRDFAELYNLGLPVAAVYFNCQRESGTGGRRM   173
       |||||||||||||||||||||||||||||||||||||||||||||||||||||
121    LLFQQLGRQTVYAPGWRQNFSTRDFAELYNLGLPVAAVYFNCQRESGTGGRRM   173
```

Supplementary Fig. S19

Sugarcane *ScFT3* sequence used for flowering analysis in transgenic *Arabidopsis*

A. Alignment of the sugarcane ScFT3 and synthetic ScFT3 coding sequences showing where synonymous substitutions were made (highlighted in the synthetic ScFT3).

B. Alignment of protein sequences showing that the amino acid sequences are exactly the same.

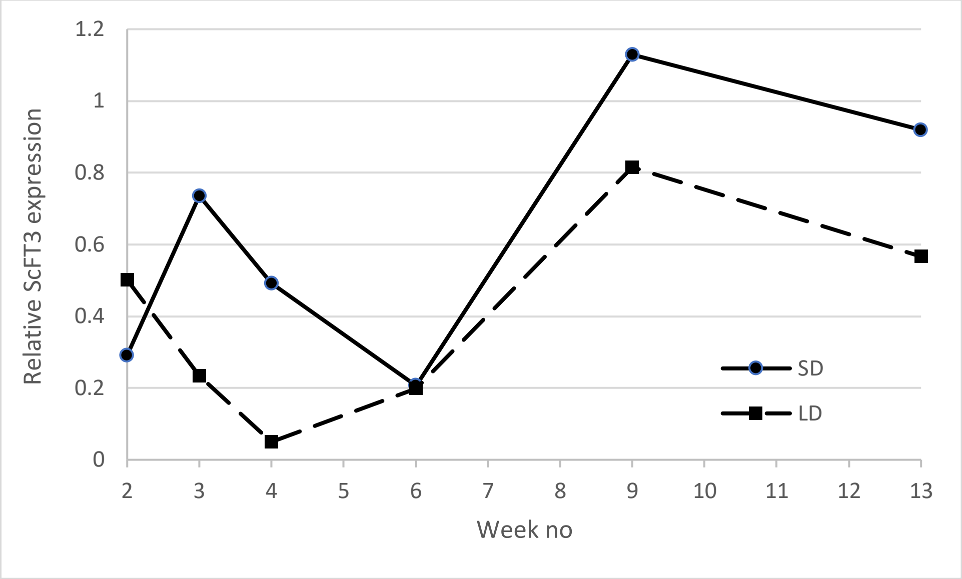

Supplementary Fig. S20

Expression of *ScFT3* in SD and LD over the developmental timecourse experiment.
Expression of *ScFT3* is calculated as the average 2^-(ΔCt) relative to two reference genes (Sc*TUB* and *ScUBQ1*). The overall difference in *ScFT3* expression in SD compared to LD, as calculated by REST, is shown in the manuscript (Fig.5b).

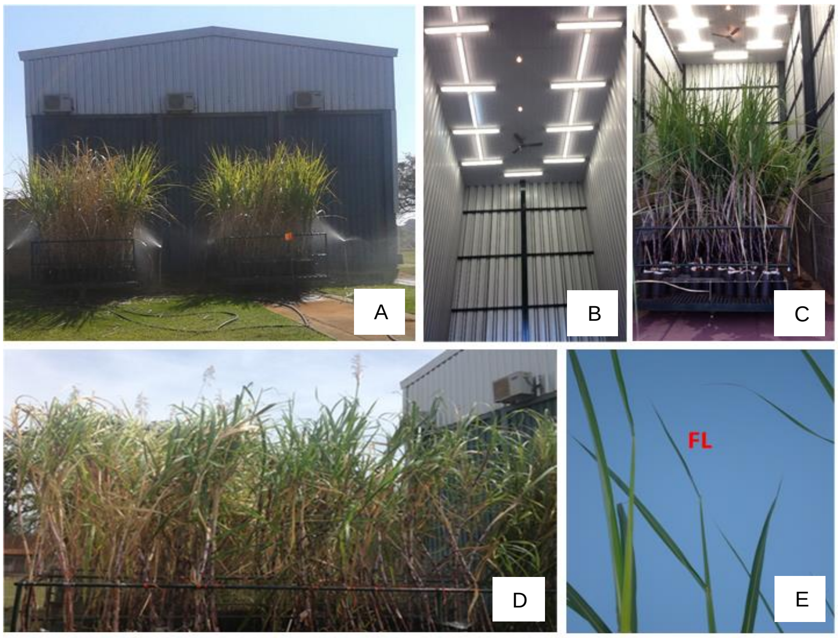

Supplementary Fig. S21

Sugarcane plants grown in the automated photoperiod facility for the developmental time course

A) outside view of the photoperiod facility with sugarcane plants planted in pots and placed in trolleys
B) and C) inside view of the photoperiod chamber
D) arrow emission on plants in the short day treatment
E) flag leaf (FL) emission

Supplementary Table S1a

Reference numbers for sugarcane loci and sequences used to construct gene tree in Fig. 4

**Sugarcane gene**	**Sugarcane Genome Hub (CIRAD) ref**	**NCBI ref**
ScFT1	N/A	MT723923.1; CA147028.1; KJ496327.1
ScFT2	Sh_219F24_contig-2_p000030<br>Sh_220O20_p000030	MW556728.1
ScFT3	Sh_251I11_p000030	MT666095.1; CA284643.1
ScFT4	Sh_246B09 _g000100<br>Sh_227M09 contig-1_g000020<br>Sh_248B09	MT666096.1
ScFT5	N/A	MT666097.1
ScFT6	N/A	MT666098.1; MN458470.1
ScFT7	Sh_252N16_g000020	MT723925.1
ScFT8	Sh_208I14_contig-2_t000010	MT723926.1
ScFT9	Sh246O02	MT723927.1
ScFT10	Sh228J16	MT723928.1
ScFT11	Sh_249H13_t000010	MT723929.1
ScFT12	Sh_210B16_p000100	MT723930.1
ScFT13	N/A	MW556729.1
ScTFL1	N/A	KJ496328.1
ScTFL2	N/A	MT978182.1
ScTFL3	N/A	MN458471.1
ScTFL4	Sh_230H12_g000030<br>Sh_202P16_g000070	MN458472.1
ScMFT1	Sh_204E12_t000040	MT723924.1
ScMFT2	Sh210E07	MT723931.1

Supplementary Table S1b

Reference numbers for loci and sequences from species other than sugarcane used to construct gene tree in Fig. 4

Sorghum	NCBI	Phytozome v12.0	Maize	NCBI	Phytozome v12.0	Rice	NCBI	Phytozome v12.0
SbFT1	XP_002436509.1	Sobic.010G045100.1	Zcn1	ABX11003.1	GRMZM2G092008	RCN1 (OsCEN)	LOC_Os11g05470.1	Os11g05470.1
SbFT2	XP_021310907.1	Sobic.003G017200.1	Zcn2	ABX11004.1	GRMZM2G156079	Hd3a (OsFTL2)	LOC_Os06g06320.1	Os06g06320.1
SbFT3	XP_002446704.1	Sobic.006G128500.1	Zcn3	ABX11005.1	GRMZM2G338454	OsFTL3 (RTF1)	LOC_Os06g06300.1	Os06g06300.1
SbFT4	XP_002454134.1	Sobic.004G206600.1	Zcn4	NM_001112772.1	GRMZM2G075081	OSFTL1	LOC_Os01g11940.1	Os01g11940.1
SbFT5	XP_002489297.1	Sobic.005G110406.1	Zcn5	NM_001112773.1	AC217051.3_FGP006	OsFTL6	LOC_Os04g41130.1	Os04g41130.1
SbFT6	XP_002462655.1	Sobic.002G262500.1	Zcn6	NM_001112774.2	GRMZM2G132880	OsFTL8	LOC_Os01g10590.1	Os01g10590.1
SbFT7	XP_021310322.1	Sobic.004G101800.1	Zcn7	ABW96230.1	GRMZM2G141756	OsFTL7	LOC_Os12g13030.1	Os12g13030.1
SbFT8	XP_002456354.1	Sobic.003G295300.1	Zcn8	NM_001112776	GRMZM2G179264	OsFTL10	LOC_Os05g44180.1	Os12g13030.1
SbFT9	XP_002438551.1	Sobic.010G164200.1	Zcn9	ABW96232.1	GRMZM2G021614	OsFTL11	LOC_Os11g18870.1	Os11g18870.1
SbFT10	XP_021302496.1	Sobic.009G199900.1	Zcn10	ABW96233.1	GRMZM2G059358	OsFTL12	LOC_Os06g35940.1	Os06g35940.1
SbFT11	XP_002443085.1	Sobic.008G082200.1	Zcn11	XP_020394352.1	GRMZM2G117057	OsFTL13	LOC_Os02g13830.1	Os02g13830.1
SbFT12	XP_002446272.1	Sobic.006G047700.1	Zcn12	ABW96235.1	GRMZM2G103666	OsFTL5	LOC_Os02g39064.1	Os02g39064.1
SbFT13-X5	XP_021311213	Sobic.003G026600.1	Zcn13	ABX11015.1	GRMZM2G108016	OsFTL4	LOC_Os09g33850.1	Os09g33850.1
SbMFT1	XP_021306050.1	Sobic.003G098800.1	Zcn14	NP_001106251.1	GRMZM2G373928	OsFTL9	LOC_Os01g54490.1	Os01g54490.1
SbMFT2	XP_002457494.1	Sobic.010G136300.1	Zcn15	AQK80489	GRMZM2G051338	OsRCN4	LOC_Os04g33570.1	Os04g33570.1
SbTFL1_1	XP_002453931.1	Sobic.004G165100.1	Zcn16	ABW96238	GRMZM2G127121	OsRCN2	LOC_Os02g32950.1	Os02g32950.1
SbTFL1_2	XP_002442808.1	Sobic.008G037300.1	Zcn17	ABW96239.1	GRMZM2G075215	OsMFT1	LOC_Os06g30370	Os06g30370.1
SbTFL1_3	XP_002450283.1	Sobic.005G038400.1	Zcn18	ABX11020	GRMZM2G158809	OsMFT2	LOC_Os01g02120	Os01g02120.1
SbTFL1_4	XP_002447782.1	Sobic.006G068300.1	Zcn19	ABW96241.1	GRMZM2G062052			
**Arabidopsis**			Zcn20	ABX11022.1	AC214791.2_FGP002	**Wheat**		
At TFL	NP_196004.1	At5G03840.1	Zcn21	ABX11023.1	GRMZM2G019993	TaFT	AY705794.1	n/a
At FT	BAA77838.1	At1G65480.1	Zcn24	ABX11024.1	GRMZM2G440005			
At TSF	AAF03937	At4G20370.1	Zcn25	ABX11025.1	GRMZM2G021560	**Barley**		
At MFT	NP_173250	At1G18100.1	Zcn26	ABW96244.1	GRMZM2G400167	HvFT	ABK91684.1	n/a

Supplementary Table S2

Sucest-Fun and Sugarcane Genome Hub contig references

FT-like genes.	Contig references	Contig Size	orientation on contig	Coverage
**ScFT1**	scga7_uti_cns_0060572	53kB	sense	Full
	scga7_uti_cns_0104067	24.5kb	sense	Full
	scga7_uti_cns_0063437	37kb	sense	Full
**ScFT2**	scga7_uti_cns_0089053	16kb	antisense	From Exon3
	scga7_uti_cns_0040609	45kb	antisense	From Exon3
	scga7_uti_cns_0032524	42kb	antisense	Full
	scga7_uti_cns_0127870	9kb	antisense	Exon 4
	scga7_uti_cns_0178011	18kb	sense	Full
	scga7_uti_cns_0126625	15kb	antisense	From Exon3
	scga7_uti_cns_0239833	29kb	antisense	Exon 4
	scga7_uti_cns_0128394	9kb	sense	Full
	scga7_uti_cns_0099432	21kb	sense	Up to Exon3
	scga7_uti_cns_0271118	12kb	sense	Full
	scga7_uti_cns_0190532	26kb	antisense	Full
	Sh_219F24_contig-2_p000030	14K	sense	Full
	Sh_220O20_p000030	10.6kb	sense	Full

**ScFT3**	scga7_uti_cns_0132301	30kb	sense	Full
	scga7_uti_cns_0311660	9.5kb	sense	No 5'UTR
	Sh_251I11 (t000030)	86kb	sense	Full
**ScFT4**	scga7_uti_cns_0115631	9kb	sense	Full
	Sh_246B099 (t000040)	145kb	sense	Full
	Sh_227M09_contig-1 (t000020)	151kb	sense	Full
**ScFT5**	scga7_uti_cns_0061975	24.8kb	antisense	Full
	scga7_uti_cns_0022519	21k	Sense	Full
	scga7_uti_cns_0010325	36k	antisense	Full
**ScFT6**	scga7_uti_cns_0347422	10.8kb	antisense	Up to Exon4
	scga7_uti_cns_0150350	41kb	sense	Full
**ScFT7**	scga7_uti_cns_0003312	35Kb	antisense	Full
	scga7_uti_cns_0125687	15.9k	sense	Full
	scga7_uti_cns_0264414	9.5kb	sense	Full
	Sh_252N16 (t000020)	115kb	sense	Full
**ScFT8**	scga7_uti_cns_0164601	36.7kb	antisense	Full
	scga7_uti_cns_0112014	15.6kb	sense	Full
	Sh_208I14_contig-2 (t000010)	13kb	antisense	Full
	Sh_220P06 (t000010)	100kb	antisense	Full
**ScFT9**	cga7_uti_cns_0418904	5.4kb	antisense	Full
	scga7_uti_cns_0408569	8.5kb	antisense	Full
	Sh_246O02 (t000130&t000110)	109kb	sense	Full
**ScFT10**	scga7_uti_cns_0012617	21.8kb	sense	Full
	scga7_uti_cns_0237025	10.5kb	sense	Full
	scga7_uti_cns_0192308	17.6kb	sense	Full
**ScFT11**	scga7_uti_cns_0036175	35Kb	sense	Full
	scga7_uti_cns_0193797	17.8Kb	sense	Full
	scga7_uti_cns_0071134	11.2kb	sense	Full
**ScFT12**	scga7_uti_cns_0245267	12kb	sense	Full
	scga7_uti_cns_0025707	15.4kb	sense	Full
	scga7_uti_cns_0197242	8.8kb	sense	Up to Exon4
**ScFT13**	scga7_uti_cns_0044937	15k	sense	Full
	scga7_uti_cns_0137098	16k	sense	Full
	scga7_unitig_337342	6k	sense	Full
	scga7_uti_cns_0137816	15k	sense	After Exon3
	scga7_unitig_325822	4k	sense	After Exon3

MFT-like genes.	Contig references	Contig Size	orientation on contig	Coverage
**ScMFT1**	scga7_uti_cns_0345261	10kb	antisense	Lack 3'
	scga7_uti_cns_0158804	10kb	sense	Full
	scga7_uti_cns_0255087	10kb	antisense	Full
	Sh_204E12 (t000040)	100kb	sense	Full
**ScMFT2**	scga7_uti_cns_0277762	7.3kb	sense	Full
	scga7_uti_cns_0169159	9.3kb	sense	Full
	scga7_uti_cns_0202450	27kb	antisense	Full
	Sh_210E07 (t000060&t000070)	141kb	antisense	Full

TFL-like genes	Contig references	Contig Size	orientation on contig	Coverage
**ScTFL1**	scga7_uti_cns_0001826	12kb	antisense	full
	scga7_uti_cns_0001701	15.8kb	antisense	full
	scga7_uti_cns_0001814	12.3kb	sense	full
	scga7_uti_cns_0138295	38k	sense	full
	scga7_uti_cns_0099427	22.4Kb	antisense	full
**ScTFL2**	scga7_uti_cns_0326520	18kb	antisense	full
	scga7_uti_cns_0269363	15.7kb	antisense	full
	scga7_uti_cns_0241826	27kb	antisense	full
	scga7_uti_cns_0204996	28Kb	antisense	full
	scga7_uti_cns_0162375	41kb	sense	full
	scga7_uti_cns_0034604	9.5kb	sense	full
	scga7_uti_cns_0034515	16kb	antisense	full
	scga7_uti_cns_0311711	6.5kb	sense	Lack 3’
**ScTFL3**	scga7_uti_cns_0421925	9kb	sense	full
	scga7_uti_cns_0353748	8.9kb	antisense	full
	scga7_uti_cns_0093573	34.7kb	antisense	full
	scga7_uti_cns_0025220	12kb	antisense	full
	scga7_unitig_28385	6.5kb	antisense	full
**ScTFL4**	scga7_uti_cns_0034052	37kb	sense	full
	scga7_uti_cns_0003021	130kb	sense	full
	scga7_uti_cns_0058760	20kb	antisense	full
	scga7_uti_cns_0001341	154kb	sense	full
	scga7_uti_cns_0129317	32.6kb	sense	full
	scga7_uti_cns_0075929	15.3kb	antisense	full
	scga7_uti_cns_0073839	10.3kb	antisense	full
	scga7_uti_cns_0012566	15kb	sense	full
	Sh_230H12 (t000030)	151kb	sense	full
	Sh_202P16 (t000070)	136kb	sense	full

Supplementary Table S3.

Relative expression (RE) of *ScFT3* over the timecourse calculated using REST, P-value (** =P<0.01), length of photoperiod (h:min:sec), and number of days after the start of the SD treatment.

**Week**	**2**	**3**	**4**	**6**	**9**	**13**
**RE**	0.674	2.542	6.527	0.892	1.369	1.779
**P-value**	0.357	0.007**	0.004**	0.758	0.526	0.030
**Photoperiod**	12:47:00	12:41:45	12:36:30	12:24:30	12:10:15	11:48:30
**Days**	8	15	22	38	57	86
